# Surface bioengineering of lanthanide nanoparticles for theranostic applications: From hydrophilic modification to multimodal imaging and therapy

**DOI:** 10.1016/j.mtbio.2026.103462

**Published:** 2026-07-18

**Authors:** Xuan Tan, Yunqiu Zhang, Shuping Wu, Dengyu Ma, Jinyu Yan, Jinwei Lu, Shikai Lin, Shiyan Li, Zhexin Hong, Shihui Jiang, Guowei Li

**Affiliations:** a School of Medical Imaging, Fujian Medical University, Fuzhou, Fujian, 350122, China; b State Key Laboratory of Structural Chemistry, Fujian Institute of Research on the Structure of Matter, Chinese Academy of Sciences, Fuzhou, Fujian, 350002, China; c University of the Chinese Academy of Sciences, Beijing, 101408, China

**Keywords:** Lanthanide nanoparticles, Surface bioengineering, Hydrophilic modification, Bio-functionalization, Theranostics

## Abstract

Lanthanide nanoparticles (LnNPs) are recognized as next-generation luminescent bioprobes due to their tunable emission spectra, long luminescence lifetimes, and high photostability. Although extensively studied, these nanomaterials have recently garnered renewed interest across biodetection, bioimaging, and therapeutics. To serve as suitable optical labels, LnNPs need to overcome inherent limitations such as hydrophobic surfaces, insufficient luminescence intensity, and limited environmental responsiveness. Accordingly, the integration of optical modulation, hydrophilic surface modification, and biofunctionality has become essential for advancing their biomedical applications. Recent surface engineering strategies have systematically addressed challenges related to hydrophilicity, colloidal stability, and biocompatibility, with substantial progress achieved. This review outlines mainstream approaches for hydrophilic surface modification and bio-functionalization of LnNPs, including ligand engineering, surface coating, and biomimetic modification. The design rationale and implementation of these methods are discussed, alongside representative applications in multimodal imaging and targeted therapy. Current challenges and future prospects in this rapidly evolving field are also summarized.

## Highlights

• A systematic overview of the strategies employed for the hydrophilic modification and bio-functionalization of lanthanide nanoparticles (LnNPs) is presented.
• The current optical and biological applications of LnNPs are elaborated in detail.
• The challenges and future prospects of LnNPs were critically analyzed and discussed.

## Graphical abstract

**Figure:**
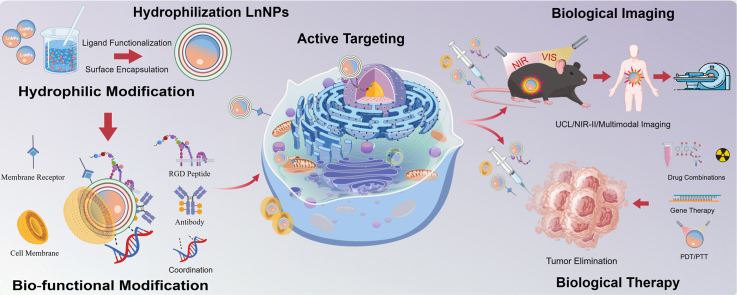

## 1 A general introduction to lanthanide-doped nanoprobes

LnNPs, especially those exhibiting up-conversion luminescence (UCL) and near-infrared region II (NIR-II) emission, represent a class of advanced optical nanomaterials with unique photophysical properties. These properties include large anti-Stokes shifts, narrow emission bandwidths, long luminescence lifetimes, high photostability, and low susceptibility to photobleaching. Collectively, these characteristics distinguish them from traditional fluorescent probes such as organic dyes and quantum dots, rendering them highly attractive for various biomedical applications, including bioimaging and diagnosis. This unique optical behavior stems from the characteristic electronic structure of lanthanides (Ln^3+^), where electron transitions within the 4*f* orbital are effectively shielded by the outer 5s^2^5p^6^ electrons, resulting in sharp atomic-like emission lines that are largely insensitive to the surrounding chemical environment. The theoretical foundation was established by the pioneering work of Dieke, Judd, Wybourne, and colleagues on the theoretical and experimental study of the 4*f*^N^ electronic structure [[1], [2], [3]]. At the application level, doped Ln^3+^ ions play multiple roles. For instance, Er^3+^, Tm^3+^, and Ho^3+^ activators can convert low-energy near-infrared light into high-energy photons, enabling up-conversion imaging free from background autofluorescence—a feature that holds great promise for luminescence bio-detection. The emission of Nd^3+^, Er^3+^, and Tm^3+^ in the NIR-II window (1000–1700 nm) provides excellent tissue penetration depth and spatial resolution for *in vivo* imaging. In addition, the sensitive response of Ln^3+^ to local lattice environments (e.g., temperature, pH) makes them ideal building blocks for constructing intelligent biosensors.

**Fig. 1:**
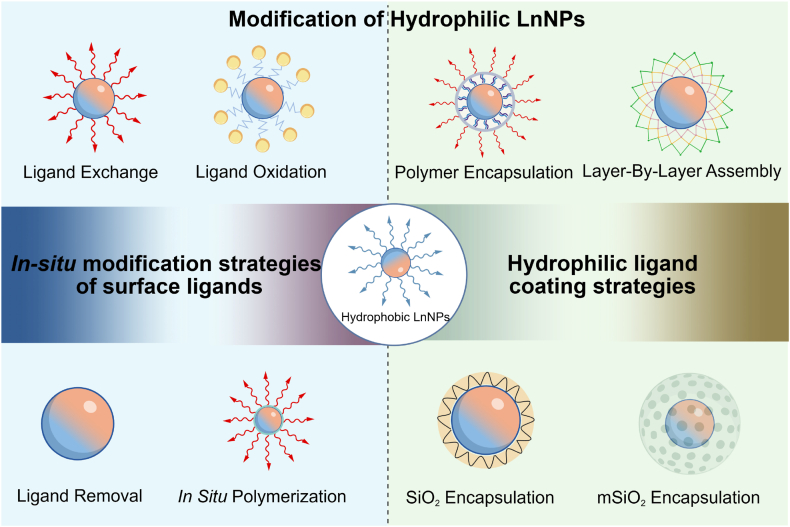
**Schematic classification of surface hydrophilic modification strategies for Ln^3+^-doped nanoparticles**. The approaches are categorized into ligand-based modification (ligand exchange, oxidation, removal, and *in situ* polymerization) and surface encapsulation (polymer encapsulation, layer-by-layer (LBL) assembly, and SiO_2_/mSiO_2_ encapsulation), corresponding to Sections 2.2, 2.3, respectively.

Advances in nanotechnology and biotechnology, particularly the development of novel synthetic methods, have directed research efforts toward the systematic integration of controlled synthesis, optical property regulation, and biomedical applications of Ln^3+^-doped nanomaterials at the nanoscale. However, although LnNPs synthesized by traditional methods (e.g., thermal decomposition and solvothermal synthesis) exhibit favorable optical properties, they are typically coated with long-chain surfactants, which restricts their dispersion to non-polar organic solvents and renders them incompatible with aqueous physiological environments [4,5]. At the same time, unmodified LnNPs themselves lack the ability to target or penetrate biological barriers. Therefore, surface bioengineering—encompassing both hydrophilic modification and bio-functionalization—has become a key bridge connecting these high-performance inorganic nanocrystals with complex biological systems. Although many studies have reviewed the chemical composition, controlled synthesis, and potential applications of Ln^3+^-doped nanoparticles in drug delivery, photodynamic therapy, and *in vivo* imaging [[6], [7], [8], [9], [10]], a systematic summary of their surface engineering strategies remains lacking. In fact, achieving favorable biocompatibility and precise bio-functionalization is essential for unleashing the unique optical properties of LnNPs and enabling their successful biomedical application. In view of this, this review aims to systematically outline surface engineering strategies for LnNPs and their application progress in biological diagnosis and therapy. Herein, the hydrophilic strategies that address the biocompatibility challenges of LnNPs are summarized, including ligand modification approaches such as ligand exchange, ligand oxidation, *in situ* polymerization, and ligand removal, as well as direct encapsulation strategies such as polymer or silica coating. These methods transform hydrophobic LnNPs into platforms that can be stably dispersed in aqueous phases, are biocompatible, and can be further functionalized (Fig. 1). Subsequently, advanced bio-functionalization techniques, including covalent coupling, click chemistry, and biomimetic membrane modification, are highlighted, which endow LnNPs with capabilities for targeted recognition, controlled drug/gene delivery, and photodynamic/photothermal therapy. Finally, this review demonstrates the specific biological applications of engineered LnNPs in multimodal imaging and targeted therapy, and discusses the prospects and challenges for their future clinical translation, along with emerging frontiers in this rapidly evolving field.

## 2 Strategies for biocompatibility modification of LnNPs

### 2.1 Strategies for surface modification to enhance biocompatibility

**Fig. 2:**
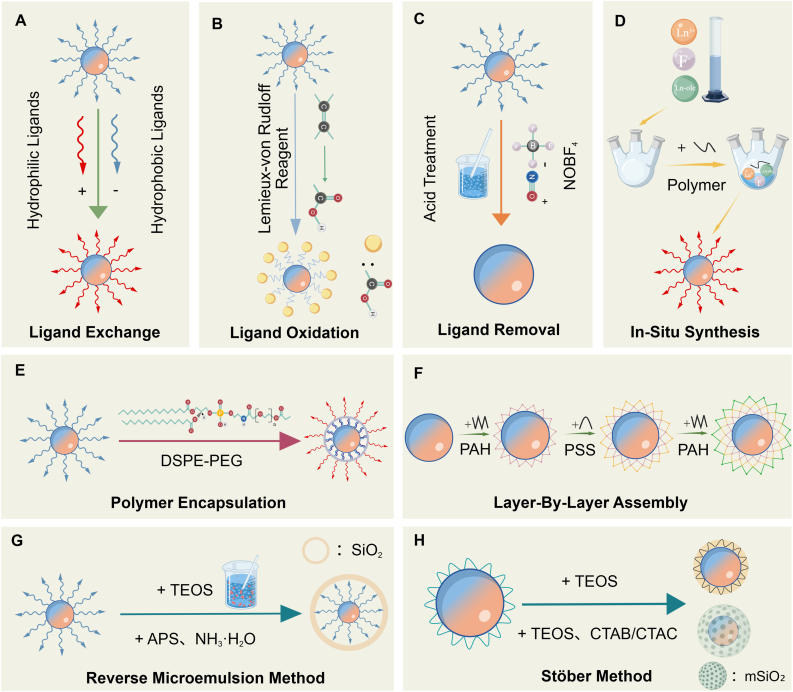
**Schematic illustration of biocompatibility modification strategies**. **A-D:** Ligand Modification, **A:** Ligand Exchange, **B:** Ligand Oxidation, **C:** Ligand Removal, **D:***In Situ* Polymerization Modification, **E-H: Surface Encapsulation Hydrophilic Modification**, **E:** Polymer Encapsulation, **F:** Layer-By-Layer Assembly, **G,H:** Surface Silanization.

Excellent biocompatibility is a prerequisite for the *in vivo* application of LnNPs. Over the past few decades, researchers have developed several methods to synthesize LnNPs, including thermal decomposition, coprecipitation, and solvothermal synthesis. However, highly monodisperse LnNPs with uniform size, shape, and excellent optical properties are predominantly synthesized in organic solvents at elevated temperatures above 200 °C This process results in a hydrophobic layer on the nanoparticle surface, typically composed of oleic acid (OA) or oleylamine (OM), which restricts their dispersion to non-polar organic solvents and renders them incompatible with highly hydrophilic physiological environments. This inherent hydrophobicity severely compromises their dispersion in aqueous media and biological buffers, leading to issues including poor biocompatibility and rapid clearance by the immune system—a primary bottleneck hindering their biomedical application and clinical translation. Therefore, scientists have developed several strategies to modify the hydrophobic surface of LnNPs (Fig. 2) before they can be further applied in various biological applications.

### 2.2 Ligand functionalization hydrophilic modification

In the past decade, various modification strategies have been developed to render hydrophobic LnNPs dispersible in water by modifying their surface ligands. These strategies include ligand exchange (Fig. 2A), ligand oxidation (Fig. 2B), ligand removal (Fig. 2C), and *in situ* polymerization (Fig. 2D) (Table 1).

**Table 1:** Summary of biocompatibility modification strategies (ligand modification).

Strategy	Reagents	Application	Advantages	Disadvantages	References
**Ligand Exchange**	Polydentate carboxylic acid ligands, amino polycarboxylic acid chelating agents, phosphate and phosphonate ligands and other ligands	Up-conversion ligation immunosorbent assay, immunocytochemistry	High stability, biocompatibility and functionalization	Complex process	[[11], [12], [13], [14], [15]]
**Ligand Oxidation**	Lemieux–von Rudloff Reagent	immunoassay for DNA	Simple process and high repeatability	Limited application system, long time, low yield	[[16], [17], [18], [19], [20], [21]]
**In-situ polymerization modification**	Ligands with polymerizable groups and rare earth ions	*In vivo* or intracellular imaging	Direct synthesis of water-soluble LnNPs	May affect the morphology of nanoparticles, size uniformity, monodispersity and luminous efficiency	[[22], [23], [24], [25], [26], [27], [28], [29], [30], [31], [32], [33], [34], [35], [36], [37], [38], [39]]
**Ligand Removal**	Hydrochloric acid/nitrosonium tetrafluoroborate (NOBF_4_)	Pretreatment for further functionalization prior to biological applications	Simple process	Not suitable for biological applications directly, luminous intensity is weak	[[40], [41], [42], [43], [44], [45], [46], [47], [48], [49], [50], [51]]

#### 2.2.1 Ligand exchange

Among the various strategies for rendering LnNPs hydrophilic, ligand exchange has emerged as a prevailing surface modification method owing to its high efficiency and versatility. The basic principle involves the use of molecules bearing strong coordination groups, which competitively displace the original hydrophobic ligands from the NPs surface. Such ideal ligands are typically bifunctional organic molecules or polymers that simultaneously possess two key functional groups: an anchoring group (e.g., carboxylate, phosphate, or thiol) that strongly coordinates to lanthanide ions, and a hydrophilic group (e.g., carboxyl, amino or hydroxyl). This bifunctional architecture enables the resulting nanoparticles (NPs) to be readily dispersed in water and further functionalized as needed (Fig. 2A).

**Fig. 3:**
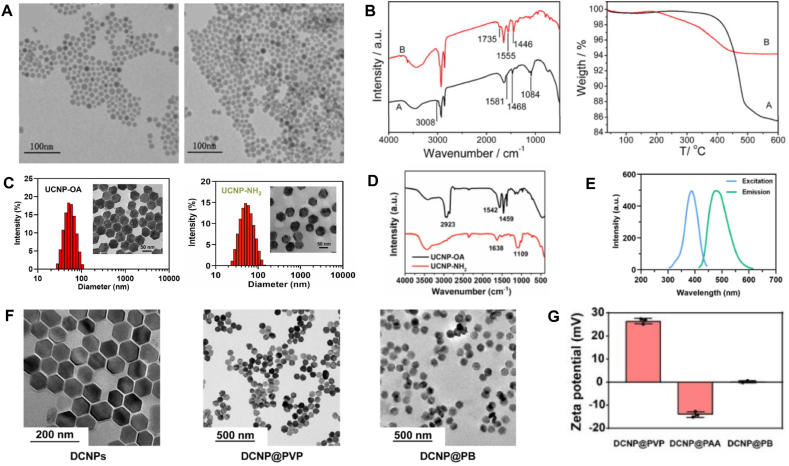
**Comprehensive characterization of surface-modified LnNPs via ligand exchange**. **A, B** Representative TEM images **(A)** and FTIR spectra and thermogravimetric analysis (TGA) curves **(B)** of NaYF_4_:Yb/Er up-conversion nanoparticles (UCNPs) before and after ligand exchange with hexanedioic acid, reproduced from Ref. [14]. The TEM images demonstrate that the nanoparticles retain their morphological integrity and dispersity after exchange, while the FTIR spectra and TGA curves confirm the successful replacement of surface oleate ligands. **C–E** Characterization of OA-capped NaYF_4_:Yb/Tm UCNPs and their AEP exchanged amino-functionalized derivatives (UCNP-NH_2_), adapted from Ref. [69]. **C** shows TEM images indicating that the morphology and size distribution remain largely unchanged after exchange, whereas **D** presents DLS measurements revealing a significant reduction in hydrodynamic size and a reversal of zeta potential from negative to positive, both consistent with successful ligand exchange. **E** displays confocal fluorescence images of cells incubated with UCNP–NH_2_, demonstrating efficient cellular uptake and bright up-conversion luminescence. **F** Characterization of DCNPs (NaGdF_4_:Nd@NaGdF_4_ core–shell nanoparticles), DCNP@PVP, and DCNP@PAA, reproduced from Ref. [56]. **G** The transition of zeta potential from positive to negative confirms the successful PAA exchange.

In 2007, Yin and colleagues reported a universal ligand exchange procedure that successfully transferred hydrophobic inorganic nanocrystals—including γ-Fe_2_O_3_, TiO_2_, and CdSe into the aqueous phase by replacing the surface OA ligands with poly(acrylic acid) (PAA) under high-temperature conditions [52]. Subsequently, numerous optimized exchange schemes have been reported [12,13,41,42,[53], [54], [55], [56], [57], [58], [59], [60], [61], [62], [63], [64], [65], [66], [67], [68], [69]]. For instance, citric acid is widely used as a mild organic acid that regulates NPs morphology through its roles as a stabilizer, complexing agent, chelator, surfactant, and capping agent in various synthetic processes [14,70,71] (Fig. 3A and B). Currently, this ligand exchange strategy is primarily employed to selectively introduce terminal functional groups, thereby yielding LnNPs bearing specific functionalities suitable for efficient biomolecule conjugation. For example, surface modification of NaYF_4_:Tm/Yb particles with 2-Aminoethyl dihydrogen phosphate (AEP) yields surface-aminated NPs [69] (Fig. 3C and D). Recent advances include a room-temperature ligand exchange method demonstrated by Wang et al., which successfully coats NaGdF_4_:Nd@NaGdF_4_@NaGdF_4_ particles with PAA using only stirring and centrifugation, thereby avoiding potential structural damage associated with high-temperature treatment [56] (Fig. 3F and G). However, this approach also has inherent drawbacks. The introduction of ligands bearing high-vibrational-frequency groups such as O–H and N–H can quench the luminescence of Ln^3+^ via non-radiative relaxation pathways, leading to reduced fluorescence (FL) intensity. For instance, after ligand exchange with AEP, the UCL intensity of KLaF_4_:Yb/Er NPs decreased to approximately one-fifth of its original value [72]. To address this issue, some studies have employed organic dye molecules with broad absorption bands as ligands to construct dye-sensitized nano-systems that compensate for the FL loss or even enhance the intrinsic luminescence [12,73].

As ligand exchange strategies have been extensively explored, the range of available molecular systems continues to expand, with increasingly diverse chemical structures and functional designs. Herein, the essential properties of several classes of ligands are summarized (Table 2). Notably, despite the wide variety of available ligands, Polyethylene glycol (PEG) functionalized with bisphosphonate or tetraphosphonate groups remains dominant in ligand exchange strategies, owing primarily to the exceptional binding stability conferred by their multiple anchoring phosphate groups.

**Table 2:** Currently the Commonly used Ligand Exchange Molecular System for LnNPs.

Ligand	Category	Remark	Reference
**Adipic acid**	Polydentate carboxylate ligands	Bidentate dicarboxylic acid	[13]
**Citric acid**	Polydentate carboxylate ligands	Tridentate and polydentate chelator	[14,70,71]
**3-Mercaptopropionic acid**	Sulfur-containing ligands	Thiol group serves as the main coordination site	[11]
**AEP**	Phosphate/phosphonate ligands	Amino phosphonate	[69,72]
**PEG dicarboxylic acid**	Polydentate carboxylate ligands	Flexible bidentate dicarboxylate	[41,60,61,65]
**PEG-phosphate**	Phosphate/phosphonate ligands	Phosphate derivative	
**PAA**	Polydentate carboxylate ligands	Polymeric polydentate carboxylate	[[54], [55], [56], [57], [58],^62^,^63^,^66^,^67^]
**PEG-PAA diblock copolymer**	Polydentate carboxylate ligands	Classified here due to the presence of the PAA block	[42]
**Polyethylenimine (PEI)**	Polyamine ligands	Rich in amine groups; cationic polymer/polydentate amine ligand	[58,68]
**PVP**	Amide-based polymeric ligands	Coordination through amide carbonyl oxygen;	[56,58,59,68]

#### 2.2.2 Ligand oxidation

To develop a simpler and more direct approach for surface modification of NPs, Lemieux and von Rudloff introduced an oxidation reagent—now known as the Lemieux–von Rudloff reagent—in the mid-20th century. This reagent cleaves alkene double bonds and oxidizes them to carboxyl groups under mild conditions [19], and it has since been widely adopted for surface modification of various nano-systems. This ligand oxidation strategy (Fig. 2B) is particularly suitable for NPs capped with long-chain unsaturated ligands. For instance, Hou et al. recently applied this oxidation route to OA-modified Fe_3_O_4_ NPs, yielding water-soluble carboxylated Fe_3_O_4_ NPs [21]. The application of ligand oxidation to LnNPs was first systematically demonstrated by Huang et al., in 2008 [16]. They successfully employed the classical Lemieux–von Rudloff reagent to oxidize the double bonds of OA ligands, yielding Ln^3+^-doped UCNPs rich in carboxyl groups that are stably dispersible in aqueous media. The surface carboxyl groups not only confer high water solubility but also enable further conjugation with various biomolecules. This strategy has been validated in diverse biological applications, including DNA sensors based on streptavidin systems [16] and anticancer drug delivery systems using Doxorubicin (DOX)-coupled NaYF_4_:Yb/Tm LnNPs [17]. Nevertheless, despite its simplicity and ease of manipulation for surface modification of hydrophobic NPs, the ligand oxidation method is applicable only to a limited subset of capping ligands that contain unsaturated carbon–carbon double bonds [4].

#### 2.2.3 Ligand removal

In contrast to surface modification strategies that introduce hydrophilic ligands through either direct or indirect means, the removal of hydrophobic ligands from LnNP surfaces—achieved using strong acid or excess ethanol under ultrasonication—offers a relatively simple and efficient route to generate Ln^3+^-exposed NPs (Fig. 2C) [15,[40], [41], [42], [43], [44], [45], [46], [47], [48], [49], [50], [51]]. For instance, Capobianco and co-workers developed a facile acid–based approach to modify OA-capped NaYF_4_:Yb/Er and NaGdF_4_:Yb/Er NPs. In their method, treatment with HCl solution at pH 4 protonated the surface oleate ligands, leading to their release from the LnNPs surface and yielding positively charged ligand-free NPs suitable for further bioconjugation [40]. The underlying mechanism involves protonation of surface-coordinated oleate ions to neutral OA molecules under acidic conditions, which weakens their coordination to Ln^3+^ and promotes their dissociation from the particle surface. Concurrently, the exposed Ln^3+^ ions may undergo further protonation to form [Ln(OH)_2_]^+^Cl^−^, conferring water dispersibility to the LnNPs. Subsequent multiple extractions enable near-complete separation of OA from the aqueous phase. This approach offers two key advantages. First, the conversion of the NPs surface to a more electropositive state enables direct electrostatic interactions with negatively charged biomolecules (e.g., nucleic acids and proteins). Second, the surface coordination sites are fully exposed, facilitating site-specific grafting of functional molecules with high loading capacity. Consequently, this strategy is particularly well-suited for constructing biological composite systems that leverage surface metal sites, such as gene delivery [49] and drug loading systems [51]. In recent years, this method has been continuously optimized toward milder reaction conditions. Recently, Suo et al. developed a protocol that combines ultrasound-assisted ethanol precipitation with dilute acid treatment to improve ligand stripping efficiency while reducing acid concentration and better preserving the morphological integrity of the LnNPs [47]. Ultrasonic treatment promotes uniform dispersion and surface perturbation of LnNPs in the acid solution, thereby accelerating OA removal and enabling the use of dilute acid, which mitigates structural damage typically associated with strong acid treatment.

In another strategy, NOBF_4_ has been employed in place of oleate to stabilize LnNPs in various polar hydrophilic media [74]. Typically, hydrophobic LnNPs in hexane are mixed with a polar solvent such as N, N-dimethylformamide (DMF) to form a two-phase mixture. Subsequent vigorous stirring removes the hydrophobic ligands from the LnNPs surface, yielding LnNPs that are monodisperse in the polar solvent with BF_4_^−^ as the counterion. It is worth noting that ligand removal, as a general method of surface engineering, is an important preprocessing for further surface modification before biological applications. However, relying solely on the surface charge of Ln^3+^ means that LnNPs after ligand removal are highly sensitive to ionic strength and medium pH, making this colloidal stability easily disrupted and thus prone to aggregation under physiological conditions. Therefore, these two methods are more widely used as important intermediate steps in further ligand modification [75], and the additional step after ligand removal is undoubtedly required to improve the stability of subsequent modification and luminescence intensity of LnNPs [15].

#### 2.2.4 *In Situ* Polymerization Modification

Although the two-step surface modification approaches described above can effectively render hydrophobic NPs hydrophilic with appropriate functional groups (e.g., carboxyl, amino) for subsequent bioconjugation, they still suffer from intrinsic limitations associated with complicated synthesis processes and tedious post-treatment procedures. To overcome these limitations, efforts have been directed toward developing more convenient one-step or one-pot methods for synthesizing a wide range of monodisperse, water-soluble, surface-functionalized, and biocompatible Ln^3+^-doped luminescent nanoparticles. *In situ* polymerization (Fig. 2D) has emerged as a one-step surface modification strategy in recent years [[22], [23], [24], [25], [26], [27], [28], [29], [30], [31], [32], [33], [34], [35], [36], [37], [38], [39]]. By initiating polymerization of surface ligands either during or after nanoparticle growth, a cross-linked polymer shell is formed around the particles, enabling *in situ* conversion from hydrophobic to hydrophilic while significantly enhancing the stability of the surface coating. Various *in situ* polymerization routes have been reported, including one-step hydrothermal [26,28], one-pot solvothermal [34], one-step thermal decomposition [33], polyol-mediated [27], and double ligand-assisted hydrothermal methods [22,39].

These methods generally follow a standard procedure: ligands bearing polymerizable groups (e.g., double bonds, epoxy groups) are first introduced into the system to adsorb onto or coordinate with the particle surface. Subsequently, polymerization is initiated under suitable conditions, forming a covalently cross-linked polymer network at the particle–solution interface. Wang et al. demonstrated the feasibility of this strategy as early as 2006 by synthesizing NaYF_4_:Ln NPs functionalized with PEI via a one-pot process [31]. Recently, Huskens et al. developed a low-temperature, short-duration (ca. 2 h) aqueous colloidal synthesis method that enables facile preparation of UCNPs with simultaneous surface functionalization [25]. In contrast, Lu et al. recently proposed a scalable synthesis scheme to directly obtain water-soluble NaYF_4_:Yb/Er LnNPs by mixing lanthanum oleate precursor, sodium fluoride, and poly(ethylene glycol) derivatives in 1-octane followed by stepwise heating to 310 °C. In this one-pot approach, nanoparticle synthesis, hydrophilic modification, and introduction of surface carboxyl groups are accomplished simultaneously [32]. Compared with traditional ligand coatings based on physical adsorption or single-site coordination, the cross-linked polymer shell formed by *in situ* polymerization is covalently attached to the particle surface, thereby exhibiting superior stability and enhanced resistance to detachment under complex physiological conditions.

### 2.3 Surface Encapsulation Hydrophilic Modification

Unlike chemical modification strategies that replace or change the original surface ligand, surface encapsulation involves the formation of a new hydrophilic coating layer around the original hydrophobic ligand outside the nanoparticle without changing it. The interactions underpinning this approach depend on the coating material used. For polymer encapsulation, amphiphilic polymers such as DSPE-PEG associate with the original hydrophobic ligand layer via hydrophobic interactions, forming a stable bilayer structure. For surface silanization, a silica shell is generated through hydrolysis and condensation of tetraethyl orthosilicate (TEOS), yielding a cross-linked Si–O–Si network covalently anchored to the nanoparticle surface. This method is expected to yield exceptionally stable, water-dispersible nanoparticles while preserving their intrinsic optical properties (Table 3).

**Table 3:** Summary of biocompatibility modification strategies (surface encapsulation).

Strategy	Reagent	Application	Advantage	defect	Reference
**Polymer Encapsulation**	Amphiphilic polymers or Amphiphilic block polymers	*In vivo* imaging	High stability, biocompatibility and functionalization	Time-consuming	[[76], [77], [78], [79], [80], [81], [82], [83], [84], [85], [86], [87], [88], [89]]
**Surface Silanization**	water, silicon sources, etc	Biomarker detection, drug loading, *in vivo* imaging		NPs increase in size or have an uncontrolled morphology	[43,59,[90], [91], [92], [93], [94], [95], [96], [97], [98], [99], [100], [101], [102], [103], [104], [105], [106], [107], [108], [109], [110], [111]]

#### 2.3.1 Polymer encapsulation

Polymer encapsulation strategies can be divided into two categories based on the interactions between the coating layer and the NPs: hydrophobicity-driven direct encapsulation (Fig. 2E) and electrostatic interaction-based layer-by-layer assembly (Fig. 2F).

**Fig. 4:**
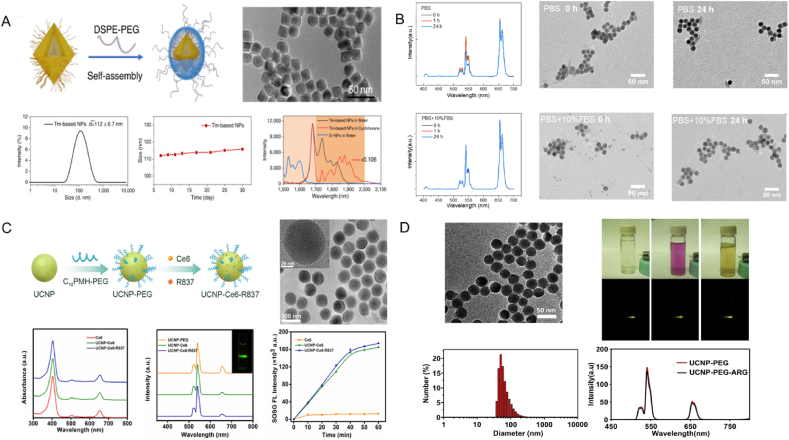
**Comprehensive characterization of amphiphilic polymer encapsulation strategies for hydrophilic modification of LnNPs**. **A** Schematic illustration of the PEGylation strategy for Tm-based downshifting NPs via hydrophobic–hydrophobic interactions between the alkyl chains of DSPE-PEG and the surface OA ligands on OA-capped Tm(0.2Er)-NPs, reproduced from Ref. [76]. The authors reported that such encapsulation enhanced colloidal stability in aqueous media and facilitated subsequent functionalization for deep-tissue bioimaging. **B** Characterization of UCNPs@DSPE-PEG shows good water dispersibility and stability [89]. **C, D** Characterizations of **C** (UCNP-Ce6 and UCNP-Ce6-R837) [85] and **D** (UCNP-PEG-ARG) [83] demonstrate that encapsulation not only enhances water dispersibility but also effectively preserves luminescence efficiency.

Hydrophobicity-based encapsulation is typically achieved using amphiphilic polymers [76,79,84,85,87,89,[112], [113], [114], [115]] or block copolymers [80], both of which possess hydrophobic and hydrophilic segments. The hydrophobic segments associate closely with the original hydrophobic ligand layer on the nanoparticle surface via hydrophobic–hydrophobic van der Waals interactions, while the hydrophilic segments extend outward, thereby rendering the originally hydrophobic NPs dispersible in aqueous media. Phospholipid-PEG derivatives (e.g., DSPE-PEG) have become widely used encapsulation materials due to their well-defined structure, favorable biocompatibility, and readily functionalizable termini (Fig. 4A). For instance, Liu and coworkers recently reported the development of a high-brightness, high-stability, neuron-targeting UCNPs@WGA probe (Fig. 4B), which was functionalized with DSPE-mPEG after core–shell–shell synthesis to achieve water solubility and biocompatibility [89]. In another study, Liu and Hong et al. demonstrated a rare earth-based NIR-II ratiometric fluorescent nanoprobe that utilizes DSPE-PEG_5000_-Mal molecules to modify the nanoparticle surface, where a dense brush layer is formed to achieve surface functionalization [112]. Numerous studies have demonstrated that such coatings significantly improve water dispersibility and colloidal stability while preserving optical properties and facilitating subsequent biological functionalization [76,78,82,83,85,88,[116], [117], [118]] (Fig. 4C–D). In particular, through rational design of amphiphilic polymers, the coating system can be endowed with stimulus-responsive characteristics, such as reactive oxygen species (ROS) responsiveness, thereby expanding its applications in theranostics. Notably, Xu et al. recently reported a composite nanosphere system that employs amphiphilic polymers to coat a luminescent core and integrate ROS-responsive functionality [87].

In contrast, LbL assembly is a surface modification method that sequentially deposits multiple material layers onto NP surfaces, primarily relying on electrostatic attraction between oppositely charged components [16]. In earlier studies, hydrophilic NPs with stable amino-enriched shells were constructed by alternately depositing positively charged polyelectrolytes (e.g., polyallylamine hydrochloride, PAH) and negatively charged polyelectrolytes (e.g., sodium polystyrene sulfonate, PSS) onto hydrophobic UCNPs surfaces [119]. In recent years, this method has been extended beyond the deposition of organic polyelectrolytes to include inorganic nanolayers such as silica, titanium dioxide, noble metal NPs, and magnetic nanoparticles—enabling the construction of organic/inorganic hybrid layers that form complex core–shell structures with specific functions [6,54,120].

#### 2.3.2 Surface silanization

Compared with polymer coatings, inorganic layers provide a more stable, inert, and readily functionalizable surface for NPs [43,84,[90], [91], [92], [93],[95], [96], [97], [98], [99],^105^,^106^,[108], [109], [110],^121^,^122^]. For example, amorphous SiO_2_ offers excellent water dispersibility, optical transparency, and biocompatibility. Moreover, the abundant silanol groups on its surface can be readily modified to introduce functional groups such as carboxyl or amino groups, facilitating subsequent coupling with biomolecules [15,107,123,124] or serving as an interface for secondary coating [99,108,120,122,[125], [126], [127], [128]]. As a result, SiO_2_ is currently widely employed in surface encapsulation strategies for LnNPs.

**Fig. 5:**
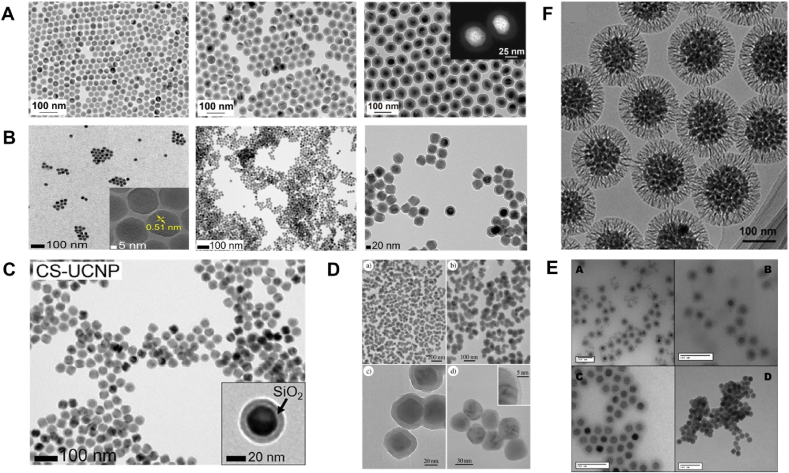
**A-C** Silica-coated LnNPs prepared via the reverse microemulsion method. As reported by the authors, silicon shells synthesized by this method are generally thicker [91,106]. **D, E** Silica-coated LnNPs synthesized by the Stöber method [101,102]. The authors reported that the silica shell is extremely thin (estimated <2 nm) and conformal. **F** TEM images of NaNdF_4_@DMS, reproduced from Ref. [95]. The nanoparticles exhibit a well-defined core–shell morphology with a mesoporous silica shell. (For interpretation of the references to colour in this figure legend, the reader is referred to the Web version of this article.)

Hydrophobic NPs are typically coated using the reverse microemulsion method (Fig. 2G) [91,95,[105], [106], [107], [108], [109], [110], [111]]. This method relies on a water-in-oil microemulsion system, where tiny water droplets are encapsulated and dispersed in the oil phase by surfactants, forming nanoscale reaction compartments. These water cores contain hydrophobic NPs, water, silicon precursors (e.g., tetraethyl orthosilicate, TEOS), ammonia, and other components. The SiO_2_ layer is formed via hydrolysis and condensation of TEOS within the water cores. Notably, because the reaction is confined to the water cores, SiO_2_ deposition and growth occur exclusively within these nanoscale compartments. Meanwhile, hydrophobic NPs tend to localize at the oil–water interface of the water cores, promoting preferential SiO_2_ deposition onto their surfaces. This confinement makes the method particularly suitable for applications that demand precise control over shell morphology and size consistency, such as in advanced biomedical fields (Fig. 5A–C). In an early study, Zhang et al. successfully synthesized NaYF_4_:Yb/Er@SiO_2_ up-conversion nanocrystals with high FL intensity, excellent aqueous dispersibility and biocompatibility [105]. They also performed the first systematic evaluation of the *in vivo* biocompatibility of these SiO_2_-coated nanocrystals in an animal model. More recently, Hlaváček et al. systematically reviewed UCNPs, summarizing their synthesis, surface modification, and bioconjugation, as well as their applications in cancer biomarker detection and imaging. Notably, they established a complete, repeatable, high-throughput process for UCNP preparation and bioconjugation [15].

In contrast to the reverse microemulsion method, the Stöber method first reported by Werner Stöber and colleagues in the 1960s enables homogeneous deposition of SiO_2_ via TEOS hydrolysis in an alcohol–water system (Fig. 2H) [103]. This strategy offers simpler operation and involves milder reaction conditions, making it particularly suitable for NPs that have undergone prior hydrophilic treatment (Fig. 5D and E). Moreover, this method is broadly applicable to various UCNPs synthesized via hydrothermal, solvothermal, or thermal decomposition routes [59,101,102,111]. In addition, the resulting colloidal NPs exhibit high stability and good monodispersity, which are among its key advantages. However, precise control over shell thickness and uniformity remains challenging. Overall, this strategy remains valuable for applications that require rapid construction of SiO_2_ shells.

Mesoporous silica (mSiO_2_) has attracted considerable attention as a drug delivery vehicle owing to its unique properties, including high surface area, large pore volume, tunable pore size with narrow distribution, and excellent chemical and thermal stability. The integration of mSiO_2_ with LnNPs to form uniform core–shell nanocomposites hold great promise for multimodal bioimaging (e.g., MRI and up-conversion imaging) [94,97], targeted anticancer drug delivery [94], and photodynamic therapy (PDT) [95,98,121]. Compared with conventional amorphous SiO_2_, the most distinctive feature of mSiO_2_ is its nanoporous structure with ordered pore arrangement and tunable pore size (typically 2–50 nm) (Figs. 2H and 5F). This structure not only provides the protective isolation and hydrophilic properties of a conventional SiO_2_ shell but also enables efficient loading of various functional molecules. These include chemotherapeutic drugs, nucleic acids [100], and photosensitizers [92,100], among others. To demonstrate its loading capacity, Wang et al. prepared mSiO_2_-coated LiYF_4_ UCNPs as donors for ROS generation in *in vitro* PDT, and employed three photosensitizers as acceptors to construct a therapeutic nanoplatform based on donor–acceptor Förster resonance energy transfer (FRET). Their results showed that the mSiO_2_-coated UCNPs achieved a high loading capacity of 30.6% (w/w) for the photosensitizer MC540, compared with OA-capped or ligand-free UCNPs [92].

### 2.4 Comparative analysis of colloidal stability of different hydrophilic modification strategies under physiological conditions

While the previous sections have detailed various hydrophilic modification strategies for LnNPs, a critical yet often underexamined parameter for their *in vivo* transformation is the colloidal stability under physiological conditions. The surface coating must not only render LnNPs water-dispersible but also prevent aggregation, protein corona formation, and premature ligand desorption in the complex biological milieu. Herein, we provide a systematic comparison of the physiological stability of the major modification strategies (Table 4).

**Table 4:** Comparative stability of hydrophilic modification strategies under physiological conditions.

Strategy	Stability in PBS (≥24 h)	Stability in Serum (≥24 h)	Resistance to Dilution	Resistance to pH Change	Long-term Storage (≥1 week, 4 °C)	Reference
**Ligand Exchange (monodentate)**	Poor (aggregates within hours)	Poor	Poor	Poor	Poor	[75,129,130]
**Ligand Exchange (multidentate)**	Good (stable for days)	Good (stable for days)	Moderate	Good	Moderate	[75,[130], [131], [132]]
**Ligand Oxidation**	Moderate (better than monodentate)	Poor (aggregates within 24 h in complete medium)	Moderate	Poor (unstable at pH < 5 or >9)	Poor	[17,18,21]
**Ligand Removal**	Very poor (instant aggregation)	Very poor	Very Poor	Very Poor	Very Poor	[43,47,49]
**In Situ Polymerization**	Excellent (>7 days)	Excellent (>7 days)	Good	Excellent (pH 4–10)	Excellent	[32,133]
**Polymer Encapsulation (amphiphilic)**	Good (24–48 h)	Good (24–48 h)	Moderate	Moderate	Moderate	[76,89]
**Surface Silanization**	Excellent (months)	Excellent (months)	Excellent	Excellent (pH 2–12)	Excellent (months)	[105,134]

Among the available surface modification strategies, ligand exchange is highly versatile, but its long-term stability hinges on the coordination strength of the anchoring group. Monodentate ligands like citric acid or those bearing high-frequency vibrational groups are readily displaced by phosphates or serum proteins, leading to aggregation within hours in PBS. In contrast, multidentate ligands such as poly(acrylic acid) or bisphosphonate-PEG exhibit much better stability owing to the chelate effect, and can maintain their hydrodynamic size for days in serum-containing media [129,130]. Cao et al. confirmed that multidentate ligand coated LnNPs exhibit superior colloidal stability in PBS [131]. Specifically, LnNPs modified with tetradentate mPEG tetraphosphonate remained colloidally stable in high concentration (200 mM) phosphate buffer for several hours before only becoming turbid, whereas samples coated with monophosphate and diphosphonate ligands precipitated out of solution within minutes. Duong et al. further confirmed the importance of multidentate ligands for colloidal stability [132]. They precisely synthesized three amphiphilic diblock copolymers with identical PEG hydrophilic blocks and anchoring blocks of equal length via RAFT polymerization, and systematically compared the colloidal stability after ligand exchange with different functional groups. UCNPs coated with phosphate groups (PMAEP) remained stable for up to one week in water, PBS, and MES buffer, whereas those coated with PAA and sulfonic acid (PAMPS) groups underwent severe aggregation within hours. In addition, Density Functional Theory (DFT) calculations revealed the adsorption energies between the three anchoring groups and lanthanide ions. the values for carboxylic and sulfonic acid groups were −77.9 and −80.0 kcal mol^−1^, respectively, while the phosphate group exhibited a binding energy that was >10 kcal/mol stronger than that of carboxylate or sulfonate groups to the UCNP surface model, in good agreement with the experimental results. These findings demonstrate that the structural and chemical properties of the ligand groups strongly affect the ligand exchange efficiency with OA-capped UCNPs, thereby influencing the colloidal stability of polymer-coated UCNPs in aqueous media. Importantly, phosphate ligands provide superior long-term stability for UCNPs owing to the complete displacement of surface OA molecules and the higher adsorption energy of phosphate groups with lanthanide ions.

In contrast to ligand exchange, ligand oxidation *in situ* modifies the surface OA ligands on LnNPs, generating soluble carboxylates directly on the surface to confer water solubility and short-term colloidal stability. Specifically, this short-term stability is subject to hydrolysis and decarboxylation of the oxidized layer at pH < 5 or >6, and significant aggregation is typically observed within 24 h in complete cell culture medium owing to non-specific protein binding [17,18,21]. A more drastic approach is complete ligand removal, which yields ligand-free LnNPs with exposed Ln^3+^ ions. While these particles are highly reactive for subsequent conjugation, they suffer from the poorest physiological stability, as the bare Ln^3+^ ions rapidly coordinate with phosphates, carbonates, or proteins, causing instantaneous aggregation in PBS or serum. Consequently, such particles are unsuitable for direct *in vivo* application unless they are immediately encapsulated by an inert shell or liposome [43,47,49].

In contrast to these exchange or removal-based methods, *in situ* polymerization constructs a covalently cross-linked polymer network around each nanoparticle. The resulting hydrophilic shell is exceptionally resistant to dilution, high salt concentrations, and pH variations between 4 and 10, with no detectable ligand leakage over several weeks. This strategy has been shown to maintain excellent colloidal stability in fetal bovine serum for more than seven days, positioning it as one of the most robust non-encapsulation strategies [32,133].

Polymer encapsulation, relies on physical rather than covalent interactions. Amphiphilic polymers such as DSPE-PEG associate with the native oleate layer via hydrophobic forces, providing good short-term stability (24–48 h) in both PBS and serum. However, prolonged incubation beyond 72 h or exposure to lipophilic molecules can disrupt the bilayer structure, leading to polymer desorption and nanoparticle aggregation [76,89]. Layer-by-layer assembled coatings offer enhanced mechanical stability through electrostatic interactions. However, at high ionic strength, they still present the risk of coating peeling [119]. An interesting recent study by Nanaho Shindo et al. reported the preparation of supramolecular nanogels via electrostatic cross-linking of cationic PEI with anionic lanthanide complexes (Ln_3_TCAS_2_) [135]. These nanogels exhibited robust colloidal stability under physiological conditions, with a very low leakage rate (0.034%). Even in high-ionic-strength PBS, the particle size remained stable in the range of 137–154 nm, no aggregation or precipitation was observed, and only slight swelling due to ionic osmotic pressure occurred.

Finally, surface silanization offers the highest colloidal stability among all the strategies discussed. Silica coatings, whether amorphous or mesoporous, form an inert and robust Si–O–Si network that maintains nanoparticle monodispersity in PBS, serum, and even at extreme pH values (2–12) for months [105,134]. However, cases where other protocols can maintain LnNPs stability for more than 6 months are still relatively rare. Interestingly, Que et al. reported a surface-coating strategy using poly(ethylene glycol)-b-poly(pentafluorophenyl methacrylate)/phosphonic acid followed by shell cross-linking with NH_2_-PEG-NH_2_ [136]. The cross-linked PEG layer provided excellent long-term colloidal stability in PBS over a temperature range of 25–60 °C. Notably, these LnNPs could endure freeze-drying without any sign of aggregation, greatly increasing storage shelf life. This strategy represents one of the few reported approaches achieving stability compatible with ≥6-month storage requirements. Although we acknowledge that systematic long-term stability data under physiologically relevant conditions remain scarce, we hope that the critical framework and comparative insights presented in this paper will inspire urgently needed future research in this direction.

### 2.5 The technological evolution of surface engineering: from functional modification to intelligent interface design

#### 2.5.1 From hydrophilic renovation to stable dispersion

Based on a thorough review of various surface modification strategies, it becomes evident that the development of surface engineering for LnNPs is not a mere accumulation of techniques. Rather, it represents a progressive evolution—from addressing isolated issues to achieving multifunctional integration, and from passive design to active adaptation. In the early stages, the core challenge was unequivocal: high-quality LnNPs necessitated high-temperature organic-phase synthesis, whereas biomedical applications demanded aqueous dispersibility. This fundamental conflict gave rise to the most basic hydrophilic modification strategies. However, when examining the progression of these methods along the temporal axis, a clear trajectory of technological iteration can be discerned.

Ligand exchange represents the earliest systematically investigated approach in surface engineering. In the initial explorations, small-molecule monodentate carboxylate ligands [14,70,71] or multidentate carboxylate ligands such as PAA [42,52,54,58] were commonly employed as general-purpose ligands for exchange. Zhang et al. successfully transferred a variety of hydrophobic inorganic nanocrystals into the aqueous phase, demonstrating the universality of ligand exchange for rendering nanocrystals hydrophilic [52]. However, researchers in the field of lanthanide nanomaterials soon recognized that these ligands coordinate to the surface Ln^3+^ ions primarily through their carboxylate groups. This coordination is disadvantaged in the presence of physiologically common ions such as PO_4_^3−^, because rare-earth ions exhibit a stronger affinity for phosphate, rendering them susceptible to displacement. Once the ligands are displaced, the loss of electrostatic repulsion and steric hindrance provided by the ligands leads to rapid nanoparticle aggregation and precipitation, accompanied by poor cellular uptake [137]. Although multidentate carboxylate ligands, owing to their cooperative chelation effect, significantly enhance the binding strength to the particle surface compared with monodentate counterparts, even if they remain intact, they may quench the up-conversion luminescence of LnNPs through nonradiative relaxation pathways. Driven by the pressing need for stability, researchers turned to PEGylated bisphosphonate or tetraphosphonate ligands [53,60,61], which exploit the strong chelating anchor provided by bisphosphonate/tetraphosphonate groups to ensure durable anchoring in complex media, while the PEG chains construct a highly hydrated hydrophilic brush layer that effectively resists nonspecific protein adsorption, thereby balancing stability and biocompatibility. It is thus evident that the demand for stability has been the core driving force behind the evolution of ligands from monodentate to multidentate architectures, and from simple small molecules to polymer/PEGylated hybrid ligands—each iteration striving more effectively to address the fundamental issue of maintaining long-term colloidal integrity of nanoparticles in physiological environments.

In addition to the well-established ligand exchange strategy, three other surface engineering approaches have been explored: oxidation, ligand removal, and *in situ* polymerization, each addressing the stability challenge from a distinct angle.

Ligand oxidation was once regarded as a more straightforward alternative. By directly oxidizing the unsaturated bonds of surface-bound OA to generate carboxyl groups, this approach circumvented the complex procedures of ligand exchange. However, this method soon revealed intrinsic limitations: it is applicable only to ligands containing C=C bonds, and the oxidized layer is prone to hydrolysis under physiological conditions, rendering its stability far inferior to that of well-designed PEGylated multidentate ligands. Once the multidentate ligand exchange approach achieved breakthroughs in stability, the operationally simpler oxidation method was consequently marginalized. In contrast, Ligand removal took an entirely different direction. When researchers discovered that the fully exposed Ln^3+^ surface, despite being extremely unstable in aqueous media, offered exceptionally high surface reactivity, they immediately recognized it as an ideal intermediate for further functionalization. This represented a significant methodological leap.

The advent of *in situ* polymerization, represented the culmination of ligand functionalization strategies. *In situ* polymerization achieved two fundamental breakthroughs: first, hydrophilization and synthesis were accomplished in a single process, eliminating post-treatment steps. second, the polymer network was anchored to the particle surface via covalent crosslinking, conferring stability far superior to that of exchange systems relying on mono- or multidentate coordination. This strategy was later extended by Lu et al. into a scalable one-pot synthesis protocol [32].

#### 2.5.2 From simple modifications to functional protection

If ligand functionalization solves hydrophilic problems, then the emergence of coating strategies addresses deeper needs: how can we bestow hydrophilicity while maximally protecting the intrinsic optical properties and colloidal stability of LnNPs?

From the perspective of technological evolution, the core advantage of encapsulation strategies lies in their non-invasiveness. Since this approach does not involve ligand exchange, the original OA layer is preserved, thereby minimizing the quenching of luminescent centers by high-frequency vibrational groups. For instance, core-shell-shell UCNPs with a NaLu_0.9_Er_0.1_F_4_@NaYbF_4_@NaLuF_4_ architecture constructed via DSPE-PEG encapsulation exhibited more than a ten-fold enhancement in single-particle UCL brightness at a low power density of 0.69 kW cm^−2^, compared to unoptimized control samples. Even at a high power density of 21.7 kW cm^−2^, an enhancement factor of 2.88 was maintained [116]. Notably, in the case of inorganic coatings, amorphous shells such as SiO_2_ can, under certain conditions, form new chemical bonds—for example, Gd-O-Si bridges—with lanthanide ions on the core surface, thereby contributing additional luminescence [138]. This effect, however, is highly dependent on shell thickness. It is generally accepted that, provided the core is fully covered, a thicker shell more effectively suppresses surface quenching, with SiO_2_ layers of approximately 8 nm, 10-12 nm being regarded as optimal [139]. Supporting this view, Shen et al. systematically investigated the influence of SiO_2_ interlayer thickness on up-conversion emission in Ag/SiO_2_/NaYF_4_:Yb/Er/Gd sandwich structures, and found that the up-conversion intensity increased continuously as the SiO_2_ layer thickness was raised from 0 to 15 nm, but decreased rapidly upon further thickening beyond 15 nm. Their study further demonstrated that reversible switching between luminescence quenching and enhancement could be achieved by varying the silica interface thickness [140].

In addition, it is worth noting that there is also a trend of functional complementarity and cross-fusion between encapsulation strategies and ligand exchange strategies. For example, SiO_2_-coated particles can introduce amino groups via (3-Aminopropyl)triethoxysilane (APTES), then target molecules are coupled via EDC/NHS coupling—essentially using the coating and ligand functionalized in series. Additionally, some studies have further enhanced SiO_2_ coating after ligand exchange to obtain a PEGylated surface, balancing colloid stability and interfacial functionality. This hybridization trend indicates that surface engineering has evolved from a single strategy selection to multi-strategy system integration.

## 3 Strategies for the bio-functionalization of LnNPs

### 3.1 Surface ligand engineering for bio-functionalization

**Fig. 6:**
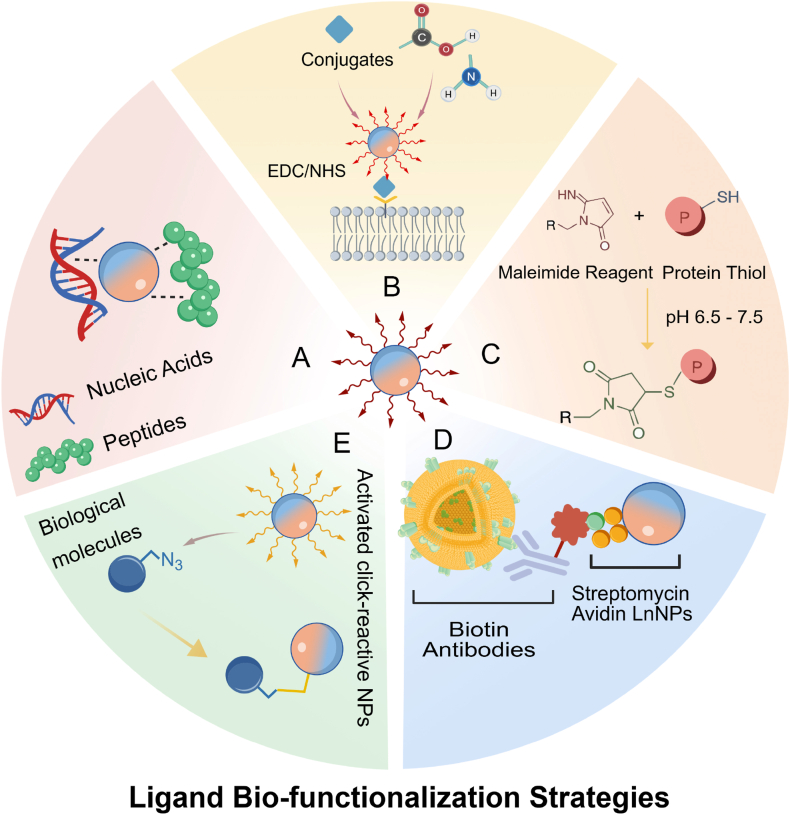
Schematic illustration of the ligand-based bio-functionalization process.

Following hydrophilic modification through ligand engineering or surface coating, LnNPs exhibit improved water dispersibility and colloidal stability, laying the foundation for their application in biological environments. However, **LnNPs** subjected solely to hydrophilic modification lack the ability to specifically interact with biological systems. To enable specific functions in complex physiological environments, functional biomolecules must be conjugated onto the LnNPs surface, a process termed bio-functionalization. This strategy is particularly relevant for applications including targeted delivery, cell penetration, biosensing, and therapy. Depending on the surface chemical properties of LnNPs (e.g., functional groups, surface charge, and ligand types), various functionalization strategies can be employed, which are broadly categorized into covalent coupling and non-covalent binding (Fig. 6, Table 5).

**Table 5:** Summary of ligand bio-functionalization strategies.

Strategy	Mechanism	Application	Advantage	defect	Reference
**Surface Coordination**	Coordination (non-covalent binding)	Metal complex connection and gene delivery platform construction	Simple steps	Colloid stability vulnerable	[47,49,51,[141], [142], [143]]
**EDC/NHS Mediated Covalent Conjugation**	Amide bond formation reaction (covalent coupling)	Targeted molecular coupling and biosensing platform construction	Mature Response High Efficiency、Reagent Accessible, Firmly Connected	Pre-modified carboxyl/amino groups are required	[11,27,[55], [56], [57],^69^,^83^,^84^,[87], [88], [89],^110^,^111^]
**Maleimide-Thiol Mediated Covalent Conjugation**	Michael addition reaction (covalent coupling)	Sulfhydryl-containing peptide targeting (e.g., angiopep-2)、Enzyme substrate peptide coupling	High reaction efficiency and specificity、few side effects	Target molecules free thiol is required	[112,144]
**Biotin-Avidin System**	Hydrogen bonding networks, van der Waals forces, and hydrophobic interactions(non-covalent binding)	Sandwich immunoassay、Cell multistaining	high stability and signal amplification potential	May trigger an immune response	[10,14,15,44,60,102,145,146]
**Click Chemistry**	Click response (covalent coupling)	Biomolecule conjugation	High reaction efficiency and specificity	Pre-finishing is complex and costly	[144,[147], [148], [149], [150]]

#### 3.1.1 Surface coordination

LnNPs with surface-exposed Ln^3+^ ions, obtained through ligand removal strategies, possess abundant and highly active metal sites that enable direct functionalization via coordination [6,8,[40], [41], [42], [43], [44], [45], [46], [47],[49], [50], [51],^75^,^151^,^152^]. Negatively charged functional groups (e.g., carboxylate, –COO^-^; phosphate, –PO_3_^2-^) in solution can strongly coordinate with these bare Ln^3+^ ions, thereby directly anchoring functional molecules onto the nanoparticle surface. For instance, Liang and colleagues treated NPs with 0.1 M HCl, followed by sonication to remove surface OA. They then directly coordinated negatively charged siRNA to the exposed Gd^3+^ on the NPs surface via the phosphate backbone, achieving efficient and stable siRNA loading while simultaneously enhancing endosomal escape [49]. Moreover, the presence of Gd^3+^ enables this platform to be readily extended into an integrated theranostic system, such as for magnetic resonance imaging-guided gene therapy. Similarly, Huang et al. constructed a near-infrared light-controlled Fenton-like reaction platform by treating OA-capped NaYF_4_:Yb/Tm UCNPs (synthesized via thermal decomposition) with a mixed HCl/ethanol solution to coordinate an iron complex bearing carboxyl/thiol groups onto the nanoparticle surface [51]. This method obviates the need for pre-introduction of coupling reagents and involves straightforward steps. It is particularly suitable for biological composite systems that directly leverage surface metal sites for loading or catalysis, although its colloidal stability is generally dependent on medium conditions. Therefore, subsequent additional steps (e.g., Liposome encapsulation) are undoubtedly necessary to improve the modification stability of LnNPs.

#### 3.1.2 EDC/NHS mediated covalent conjugation

Among the various covalent coupling methods, carbodiimide-mediated amide bond formation—typically employing 1-ethyl-3-(3-dimethylaminopropyl) carbodiimide (EDC) and N-hydroxysuccinimide (NHS) has become a widely used strategy for LnNPs bio-functionalization owing to its well-established reaction conditions, high efficiency, and readily available reagents. The reaction typically begins with pre-modified carboxyl groups (–COOH) on the NPs surface. EDC first reacts with these carboxyl groups to form an unstable O-acylisourea intermediate, which is subsequently converted to a more reactive NHS ester upon interaction with NHS. This active ester then undergoes nucleophilic substitution with primary amino groups (–NH_2_) on biomolecules (e.g., antibodies, peptides, folic acid), forming a stable amide bond and achieving covalent immobilization of the biomolecules. Alternatively, if the NPs surface is pre-modified with amino groups, the carboxyl groups on biomolecules can be activated by EDC/NHS and subsequently coupled to the surface amino groups.

Carboxyl groups on NP surfaces can be introduced via ligand exchange [27,55,56], ligand oxidation, or amphiphilic polymer coating [88,89,153]. Amino groups can be introduced through ligand exchange using AEP as a ligand [69] or via APTES modification after silanization [154]. These functional groups provide the necessary reaction sites for EDC/NHS coupling. Coupling targeting molecules onto these functionalized surfaces represents a common strategy to endow NPs with active targeting capability (Table 6). Such modified NPs can efficiently target the cell membrane, cytoplasm, mitochondria, or other intracellular organelles owing to their surface-anchored targeting ligands. For instance, Hong's group demonstrated good cell targeting ability with DSPE-PEG-FA-coated Lu-YNP@FA nanoparticles [153], as well as with DSPE-PEG-anti-HER2-coated ^177^Lu-LnNRP in a separate study [156]. Notably, the DSPE-PEG-GA-coated UCNP platform developed by Dong and collegues enables direct mitochondrial targeting [113]. In addition, this method has been widely employed to construct biosensing platforms by coupling recognition units to NPs surface for highly sensitive detection of biomarkers [107,157,158].

**Table 6:** The EDC/NHS method enables the coupling of receptor molecules.

Molecule	Role position	Application	Reference
**Folic acid (FA)**	Folate receptors (membrane receptors)	Tumor targeting	[27,69,[153], [154], [155]]
**Anti-HER2**	HER2 receptors (membrane receptors)	Tumor targeting	[156]
**amino-modified thrombin aptamer**	Thrombin-aptamer complex	FRET system	[55]
**cDNA**	cDNA-aptamer complex	FRET system	[57]
**AlOCPc**	/	Multimodal imaging and photodynamic therapy	[110]
**NH**_**2**_**-PEG3400-COOH、D-SP5和UEA-I**	Overexpressed glycosylated epitopes	Tumor targeting and optical imaging	[111]
**Oligoarginine**	Cell membranes	Stem cell labeling and *in vivo* tracing	[83]
**Croconaine**	Lysosomes	Promote tumor death	[69]

#### 3.1.3 Maleimide-thiol mediated covalent conjugation

Maleimide–mercapto chemistry has emerged as a vital bioorthogonal tool for achieving higher specificity and biocompatibility in surface coupling strategies. This chemistry enables the formation of stable thioether bonds via efficient Michael addition of maleimide groups to sulfhydryl groups under neutral to weakly basic conditions [144]. Compared with traditional EDC/NHS conjugation, this reaction offers higher specificity and fewer side reactions with common biological functional groups, contributing to product homogeneity. Additionally, it proceeds under mild conditions within the physiological pH range, which is advantageous for preserving biomolecular conformation and activity. For instance, Li et al. designed a maleimide-functionalized dye-brush polymer (Dye-BP) to modify OA-coated UCNPs via ligand exchange, followed by covalent conjugation of the targeting peptide angiopep-2 to the NPs surface using the maleimide–thiol reaction to achieve glioma targeting [73]. In a recent study, Liu and Hong et al. developed a rare-earth-based NIR-II ratiometric fluorescent nanoprobe featuring DSPE-PEG_5000_-Mal on the NPs surface. Using this chemistry, they conjugated a cysteine-containing granzyme B substrate peptide to the probe, enabling real-time, non-invasive imaging of enzyme activity *in vivo* [112]. Notably, for molecules lacking native sulfhydryl groups, genetic engineering or chemical derivatization is required to introduce such groups for the reaction to proceed, which adds a degree of operational complexity.

#### 3.1.4 Biotin-avidin system

The conjugation constant between biotin and avidin (or streptavidin) is exceptionally high, reaching ∼10^15^ M^−1^, rendering the interaction essentially irreversible. Thus, as a universal bridging platform exploiting this exceptionally strong non-covalent interaction, the biotin–avidin system offers unparalleled binding stability for NPs functionalization, along with signal amplification potential. The unique value of this system has been demonstrated across diverse biological applications. For instance, it has been widely employed to construct highly sensitive sandwich immunoassay platforms for *in vitro* diagnosis [15,60,146], and to enable highly flexible multiplexed staining in cell labeling and imaging [10]. Furthermore, the modular nature of the biotin–avidin system facilitates the rapid assembly of drug-loaded NPs with biotinylated ligands in targeted drug delivery [44]. However, the introduction of foreign proteins may trigger immune responses, and mitigating this immunogenicity remains a critical challenge.

#### 3.1.5 Click chemistry

**Fig. 7:**
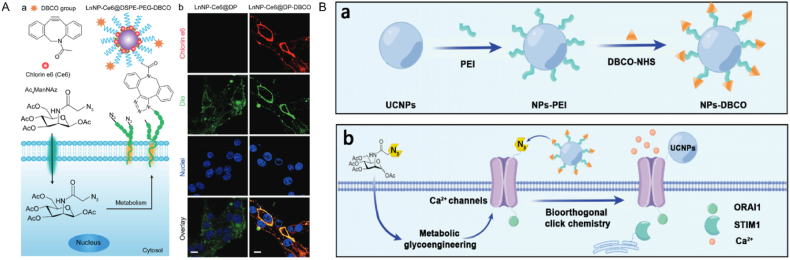
**A** Copper-free click reaction-based *in vitro* luminescence imaging and Schematic of cell labeling using polymer-coated LnNPs (LnNP-Ce6@DSPE-PEG-DBCO) [149]. **B** Schematic diagrams. (a) Design of lanthanide nanothermometer. (b) Cell membrane labeling processes [148].

Click chemistry, specifically copper(I)-catalyzed azide–alkyne cycloaddition (CuAAC) [144] and copper-free strain-promoted azide–alkyne cycloaddition (SPAAC) [[148], [149], [150]] (Fig. 7A), has become an effective tool for the surface modification of LnNPs, attributed to its high reaction efficiency, excellent specificity, and favorable biocompatibility. Currently, the most common strategy is the “functionalize-before-click” approach. In this strategy, hydrophobic LnNPs are first synthesized and then coated with amphiphilic molecules bearing click-functional groups (e.g., azido or alkynyl, as in DSPE-PEG_2000_-N_3_) or with polymeric brushes rich in such groups constructed via surface polymerization. Subsequently, these NPs can undergo cycloaddition with biomolecules (e.g., antibodies, peptides, folate, nucleic acid aptamers) carrying complementary functional groups under appropriate conditions. The feasibility of this approach has been demonstrated in several studies. For instance, Daniel Horák et al. [41] and Ren et al. [73] showed that targeting peptides such as iRGD, cRGD, or Angiopep-2 can be directly attached to LnNPs surfaces via this method, thereby endowing them with targeting or membrane-penetrating capabilities. In other specialized applications, such as the *in situ* temperature mapping strategy on immune cell membranes first reported by Yang et al., UCNPs were functionalized with dibenzocyclooctene (DBCO) groups to selectively react with bioorthogonal azide groups introduced on the target cell surface, enabling specific cell membrane labeling and *in situ* temperature sensing [148] (Fig. 7B). The “click-before-functionalization” strategy is relatively uncommon. This approach typically involves pre-linking small-molecule ligands bearing clickable groups to biomolecules, followed by ligand exchange with the LnNPs [147]. The Cu(I) catalyst used in classical CuAAC reactions may exhibit some cytotoxicity and can quench the LnNPs, limiting its *in vivo* applicability. In contrast, while SPAAC avoids the use of copper catalysts, it suffers from higher reagent costs and slower reaction kinetics. Overall, the pre-introduction of clickable functional groups adds synthetic complexity. Although other conjugation methods may lack the specificity of click chemistry, they offer simpler operation without the need for complex chemical premodification and therefore remain viable alternatives in certain applications.

### 3.2 Functional modification strategies of LnNPs after surface encapsulation

Unlike direct molecular-scale modification on LnNPs surfaces, surface encapsulation strategies utilize materials such as silica, polymers, or biofilms to completely encase the hydrophobic LnNPs core. This approach generates a homogeneous interface that enables subsequent functionalization while simultaneously conferring hydrophilicity. Although the subsequent biomolecular modification steps are largely analogous, the robust and well-defined shell layer confers distinct advantageous properties to the LnNPs, which constitutes a notable advantage of this strategy.

#### 3.2.1 Surface modification after silanization

**Fig. 8:**
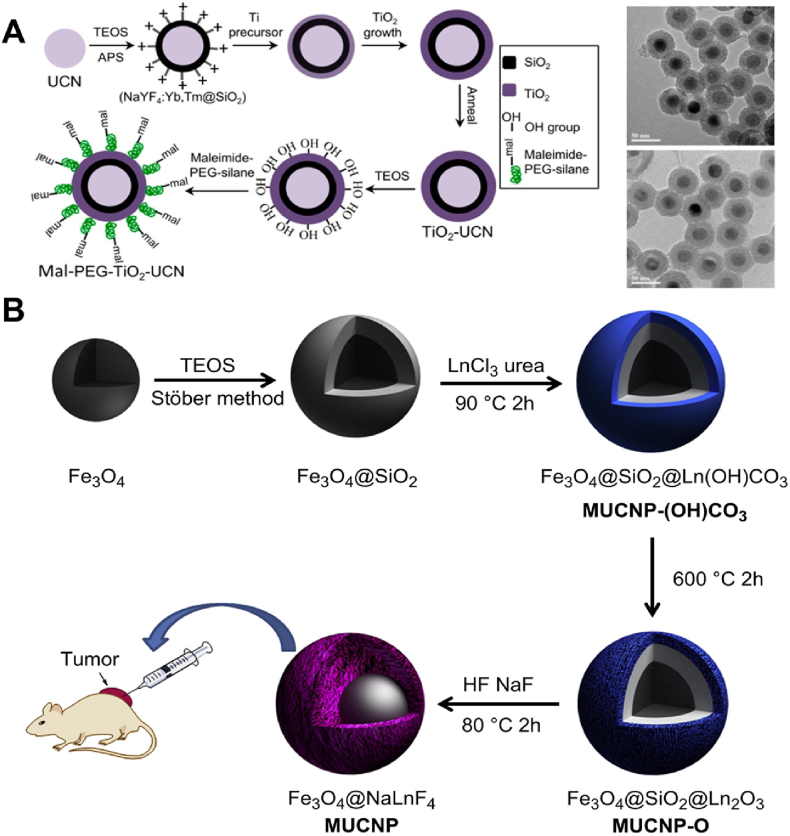
**Schematic illustration of surface functionalization strategies after silanization of LnNPs**. A Further deposition of the TiO_2_ layer is beneficial for photocatalytic PDT. **B** Schematic illustration of inverse core–shell composite system.

The surface of SiO_2_ or mSiO_2_ layers is rich in silanol (–SiOH) groups, which react readily with a variety of silane coupling agents, allowing for the incorporation of diverse active functional groups, including –NH_2_, –COOH, –SH, and maleimide, among others. For instance, treatment with APTES is frequently employed to introduce abundant –NH_2_ onto NP surfaces [95,100,110], providing a foundation for subsequent bioconjugation. This silanization–functionalization–coupling strategy facilitates the immobilization of targeting moieties—such as antibodies, peptides, and nucleic acid aptamers—onto NPs surfaces, thereby potentially enhancing their targeting capability in biological environments. As an example, surface amino-modified SiO_2_@LnNPs can be covalently conjugated to carboxyl-containing targeting molecules (e.g., folic acid, antibody fragments) via EDC/NHS chemistry [107]. Beyond serving as a platform for covalent biomolecule attachment, the silanized layer may also serve as an active interface for the secondary deposition of inorganic layers, enabling the construction of inorganic–inorganic composite core–shell structures with integrated multifunctional properties [99,108,126,127,159]. For example, depositing TiO_2_ or ZnO onto the SiO_2_ layer introduces photocatalytic activity that may be applicable to photodynamic therapy (Fig. 8A) [126]. Similarly, the deposition of a MnO_2_ layer allows for tumor microenvironment-responsive degradation, with the potential release of Mn^2+^ ions. Such Mn^2+^ ions may subsequently participate in the activation of the cGAS–STING signaling pathway following cellular internalization and may also function as T_1_-weighted contrast agents for magnetic resonance imaging (MRI) [159,160]. Furthermore, the deposition of noble metal nanoshells (e.g., Au, Ag) can impart plasmonic resonance effects on the material surface, which may be exploited in applications such as surface-enhanced Raman scattering (SERS) imaging or photothermal therapy. Notably, through a reverse design strategy, LnNPs may also be deposited as an outer layer onto SiO_2_ surfaces (Fig. 8B), forming an inverse core–shell composite system [122].

#### 3.2.2 Membrane coating

**Fig. 9:**
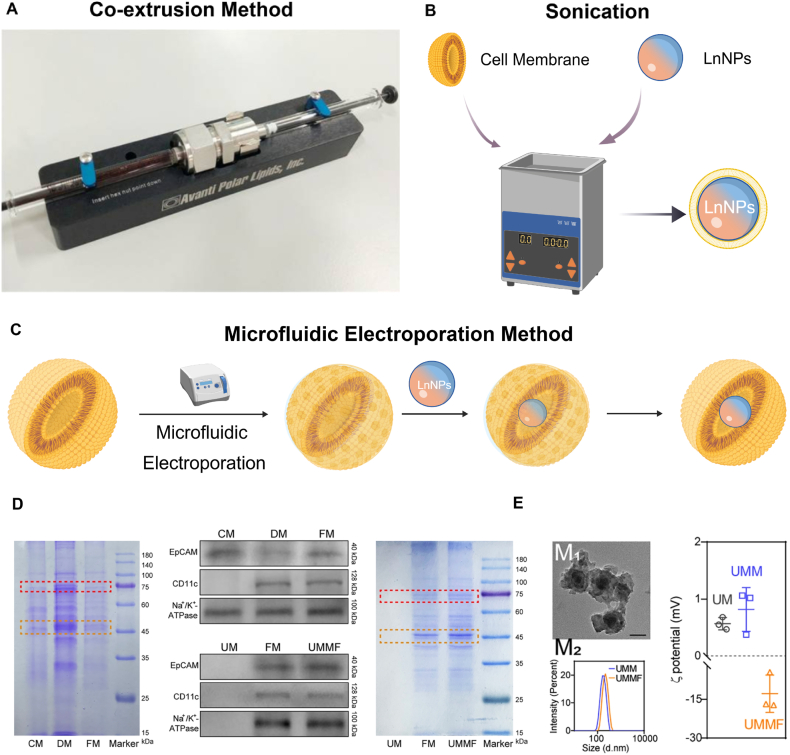
**Schematic illustration of three commonly employed methods for cell membrane coating (A**–**C) along with representative characterization results (D–E)**. **A** Co-extrusion, which is relatively mild on membrane proteins but suffers from low throughput and potential loss of membrane material during multiple extrusion cycles [170]. **B** Sonication, which is simple and rapid, yet the cavitation effect may damage membrane proteins and lipid structures, thereby compromising functional integrity. **C** Microfluidic electroporation, which offers high controllability and efficiency, but remains limited by high equipment costs and modest production scalability. **D** SDS-PAGE and Western blotting confirm the presence of membrane proteins, but these techniques cannot assess protein topological orientation or functional activity, nor can they distinguish between fully and partially coated nanoparticles. **E** TEM and zeta potential measurements are commonly used to verify successful coating; however, TEM may produce artifacts that suggest complete coverage, while zeta potential only provides ensemble-averaged information. As discussed in Section 3.2.2, these methods collectively fall short of guaranteeing coating uniformity and integrity, and practical challenges such as protein denaturation, incomplete coating, batch-to-batch variability, scale-up difficulties, and sterilization remain critical hurdles for clinical translation [171].

In contrast to inorganic coating strategies, biofilm coating represents a distinct class of biomimetic nanotechnology. This approach involves extracting the lipid bilayer from natural cell membranes and coating it onto NPs surfaces, thereby enabling nanoparticles to acquire the complex surface functionalities and biological characteristics of the source cells [161,162]. Among the reported techniques, three methods (Table 7) are most frequently employed: co-extrusion (Fig. 9A), ultrasonication (Fig. 9B), and microfluidic electroporation (Fig. 9C).

**Table 7:** Membrane coating strategies.

Strategy	Advantage	defect	Reference
**Co-extrusion**	High efficiency Good reproducibility between batches Membrane protein activity was well preserved	Limited by the filter pore size May cause damage to membrane proteins Multiple extrusion may cause sample dilution	[[163], [164], [165]]
**Sonication**	Simple and fast Low equipment requirements	Cavitation effects may damage membrane protein activity Difficulty in reproducing Only suitable for small-scale preparation and difficult to scale up production	[163,[166], [167], [168]]
**Microfluidic Electroporation**	Continuous process Highly controlled Extremely efficient	High equipment cost Complex parameters	[161,162,169]

Cell membrane-coated LnNPs retain the optical properties of LnNPs while integrating various biological functions of cell membranes, including immune evasion, targeted recognition, and favorable biocompatibility [161,[172], [173], [174], [175], [176], [177], [178], [179]]. Following successful coating, systematic characterization is typically required to confirm the integrity of the membrane shell. Transmission electron microscopy can reveal a distinct core–shell structure, dynamic light scattering can indicate an increase in hydrodynamic diameter, and a shift in zeta potential from positive to negative may serve as an indicator of successful encapsulation (Fig. 9D). Furthermore, the preservation of key membrane proteins can be verified by techniques such as SDS-PAGE and Western blotting (Fig. 9E) [170,176,[180], [181], [182], [183], [184]].

Since its first systematic report by Zhang et al., in 2011 [181], cell membrane coating technology has advanced rapidly. Erythrocyte membranes, owing to their ready availability, low immunogenicity, and extended circulation time *in vivo* (approximately 120 days), have emerged as a common template for constructing long-circulating nanocarriers. Subsequently, the repertoire of membrane sources has expanded to include erythrocyte membranes [170,176,[179], [180], [181], [182], [183], [184]], tumor cell membranes [161,172,177,178], macrophage membranes [174,175,185], stem cell membranes [186], and hybrid membranes derived from the fusion of different cell types [171,187] (Table 8). These biomimetic platforms combine the superior optical properties of LnNPs with the complex biological functionalities of cell membranes, and they have shown potential in various biomedical applications. Such applications include targeted cancer therapy and imaging [172], immune regulation and vaccine development, immune evasion and long circulation [176], as well as inflammation targeting and therapy [174,175]. Recent efforts in this field have increasingly focused on enhancing functional precision and structural complexity. For instance, Nie and colleagues developed a dual-targeting system designated Apt-pM, which is capable of simultaneously targeting inflammatory sites and specific pathogens. This system was constructed by coating drug-loaded NPs with membranes derived from lipopolysaccharide (LPS)-pretreated macrophages and embedding an aptamer (F23) targeting *Pseudomonas aeruginosa* onto the membrane [185]. In another study, Chen et al. employed PEG-mediated cell fusion to generate fusion membranes (FM) from 4T1 tumor cells and dendritic cells, thereby integrating homologous targeting and immune activation functions [171]. Collectively, these advances underscore the potential of cell membrane coating technology in the development of smart nanocarrier systems for drug delivery.

**Table 8:** Membrane source and function.

Membrane Type	Core Feature	Reference
**Red Blood Cell Membrane**	Immune escape, Long-lasting cycle, Enhanced biocompatibility	[170,176,[179], [180], [181], [182], [183], [184]]
**Macrophage Membrane**	Target inflammation, Target the tumor microenvironment	[175,185]
**Cancer Cell Membrane**	Homologous targetin, Induce anti-tumor immunity	[12,161,172,177,178]
**Stem Cell Membrane**	Tumor Homing, Wound site chemotaxis	[186]
**Hybrid Membrane**	Integrate multiple functions (such as immune escape and active targeting)	[171,187]

However, the clinical translation of this biomimetic strategy faces a cascade of interconnected hurdles. First, the biological raw materials are inherently variable, as membrane protein expression profiles fluctuate with passage number and culture conditions, challenging batch-to-batch consistency [188]. During the coating process, mechanical stress from extrusion or sonication not only causes protein loss but may also invert their topological orientation, rendering key functional moieties such as the "don't eat me" signal of CD47 inactive. Conventional SDS-PAGE or Western blotting merely confirms protein presence and cannot detect such misorientation, which has substantial practical implications because it may lead to false-positive results in functional assays [189,190]. More critically, even when proteins are preserved, the coating is rarely complete. Liu et al. recently reported that up to 90% of biomimetic NPs are only partially coated rather than perfectly enveloped [191]. The degree of membrane coating directly influences the internalization mechanism of NPs. Highly coated NPs (≥50%) enter cells individually, whereas those with low coating coverage (<50%) require aggregation for internalization. Beyond these structural defects, manufacturing obstacles loom large. Current protocols are labor-intensive and operate at milligram scales. Microfluidic electroporation offers only modest throughput at high cost, and no standardized GMP-compatible process currently exists. This also indirectly renders aseptic processing and 0.22 μm filtration the only viable but expensive alternatives for preparing biomimetic LnNPs [192], as conventional terminal sterilization methods, including autoclaving, ultraviolet, or gamma irradiation, are highly likely to disrupt the lipid bilayer. Ultimately, even well-coated sterile nanoparticles may trigger anti-membrane antibodies or complement activation upon repeated dosing, and these risks remain underexplored in preclinical models [193,194].

Liposome coating represents another important biomimetic coating strategy. While similar to cell membrane coating in terms of structural principles, it differs in composition sources, functional designability, and preparation complexity (Table 9). Liposomes typically form through the spontaneous assembly of amphiphilic phospholipids (e.g., sphingomyelin, glycerophospholipids) in aqueous media, and may consist of single or multiple lipid bilayers encapsulating internal aqueous compartments. This coating strategy improves the biocompatibility of the particles and offers the possibility to enhance the intrinsic optical properties of LnNPs. For instance, the electroactive outer membrane of *Shewanella oneidensis* MR-1, which is rich in cytochrome *c*, was utilized to construct hybrid liposomes coated with UCNPs via membrane fusion. This approach achieved UCL enhancement independent of conventional photosensitizers, offering insights into energy transfer mechanisms [195]. Conversely, the membrane-like structure of liposomes enables surface modification with various targeting molecules to achieve active targeting functions. For instance, antibodies, peptides, or small-molecule ligands can be covalently conjugated onto liposome surfaces to construct targeted delivery systems, thereby prolonging particle retention at lesion sites and promoting cellular endocytosis. In a recent study, Kawakami and colleagues reported an Fc region-mediated specific adsorption method for directional antibody immobilization on lipid nanoparticle (LNP) surfaces, offering an alternative to conventional random conjugation approaches [196]. The resulting functionalized lipids exhibit favorable water dispersibility, can be incorporated into pre-formed LNPs via gentle post-insertion, and allow facile modification through simple mixing with antibodies, demonstrating high versatility. Collectively, these advances illustrate that liposome coating has evolved from a simple carrier loading strategy into an intelligent platform capable of integrating multiple functions, including targeting, responsive drug delivery, and energy transduction [40,51,195,197].

**Table 9:** Differences and similarities between liposome and cell membrane encapsulation.

Characteristic	Cell Membrane Coating	Liposomal Coating
**Designability and functionality**	Function depends on the source cells Complex additional modifications	Highly flexible Easy to chemically modify
**Industrial quality control**	Complex process High risk of variation between batches	Mature technology Easy to scale up Batch uniformity
**Drug loading capacity**	Mainly depends on kernel or membrane tessellation	Can simultaneously carry water/fat soluble drugs
**Biocompatible/Long Cycle**	Natural immune escape	Dependent on PEGylation, but may still be identified
**Targeting capabilities**	Natural intrinsic targeting ability	Dependent active modification
**Stability and storage**	Harsh storage conditions	Stable structure, freeze-dried, long storage period
**Technical complexity**	Relatively high	Relatively low

## 4 Diverse biological applications of lanthanide nanoprobes

Systematic surface modification engineering has effectively addressed the biocompatibility limitations of LnNPs and established a foundation for their functional integration. Owing to their customizable interface chemistry and favorable biological interface properties, these nanomaterials have demonstrated considerable potential across diverse biomedical fields. Within the realm of biological imaging, particular attention is given to their deep-tissue imaging capability under near-infrared excitation, the superior spatial resolution afforded by NIR-II emission, and the integration of multimodal imaging strategies. Regarding targeted delivery and therapy, this review examines the mechanisms of targeted modification, as well as the design principles and therapeutic efficacy of drug and gene delivery systems. By reviewing these representative applications, this section aims to elucidate the intrinsic relationship between surface engineering and biological function, provide guidance for the rational design of next-generation lanthanide nanobioprobes with enhanced performance, and facilitate the development of integrated theranostic nanoplatforms that combine imaging, targeting, therapy, and feedback capabilities.

### 4.1 Biological imaging

Owing to their unique photophysical properties, LnNPs have emerged as an important complement to conventional fluorescent dyes and quantum dots. Through surface engineering optimization—including hydrophilic modification, polymer encapsulation, and targeted ligand conjugation—the luminescence performance, colloidal stability, and biodistribution characteristics of LnNPs have been significantly enhanced, establishing a foundation for their application in biological imaging. Current applications leveraging these properties primarily encompass three categories: near-infrared-excited up-conversion luminescence imaging, which offers low background interference and is well-suited for deep-tissue detection; NIR-II imaging, which achieves superior tissue penetration depth and spatial resolution through energy level modulation and dye sensitization; and multimodal imaging, which capitalizes on the synergy and information complementarity of multiple imaging modalities.

#### 4.1.1 UCL imaging

**Fig. 10:**
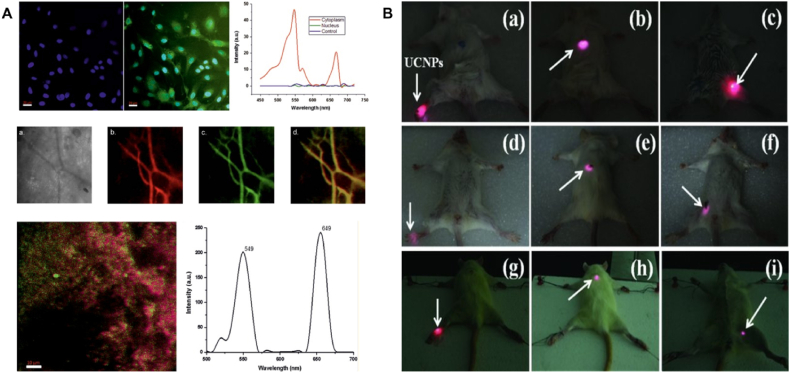
**Representative UCL imaging applications of LnNPs across different biological scales, from *in vitro* cellular imaging to *in vivo* deep-tissue visualization**. **A** Multiphoton images of DAPI‐stained endothelial cells with low concentration of NPs (0.3 ng/mL) added [198]. **B** shows *in vivo* UCL animal imaging of a tumor-bearing mouse model [118].

Unlike conventional down-conversion fluorescent probes, UCL imaging relies on near-infrared excitation, which effectively minimizes autofluorescence interference in biological tissues. This modality offers a high signal-to-noise ratio, favorable tissue penetration depth, and reduced photodamage to samples. Owing to these distinctive advantages, Ln-doped UCNPs have emerged as an important class of luminescent optical probes. Following surface engineering optimization, Ln-doped UCNPs have been widely employed in high-contrast imaging studies, ranging from cell labeling to deep-tissue *in vivo* imaging [151] (Fig. 10). The first demonstration of up-conversion bioimaging was reported by Zijlmans et al., in 1999 using submicron Y_2_O_2_S:Yb/Tm particles (0.2–0.4 μm) [199]. Subsequently, other oxysulfide and oxide nanomaterials, such as Y_2_O_3_:Yb/Er and Gd_2_O_3_:Yb/Er, have also been successfully applied in UCL bioimaging [198,200]. This field has advanced particularly rapidly over the past decade. In terms of activators, Er^3+^, Tm^3+^, and Ho^3+^ are commonly employed to achieve UCL imaging [8]. Regarding matrix materials, fluorides have attracted considerable attention owing to their low phonon energy and reduced excited-state quenching. For instance, NaYF_4_ and NaGdF_4_ are regarded as highly effective UCL hosts due to their favorable crystal structure and photophysical properties. Their hexagonal phases provide a suitable crystal field environment for doped lanthanide ions and suppress non-radiative relaxation through efficient energy transfer, thereby enabling high UCL efficiency. Consequently, up-conversion nanoprobes based on NaYF_4_ and NaGdF_4_ hosts have been widely utilized in biomedical research. These applications range from *in vitro* cell imaging (Fig. 10A) with high signal-to-noise ratio and excellent sensitivity to *in vivo* animal imaging (Fig. 10B) capable of deep tissue penetration and therapeutic guidance [29,118,120,125,[201], [202], [203]]**.**

#### 4.1.2 NIR-II imaging

**Fig. 11:**
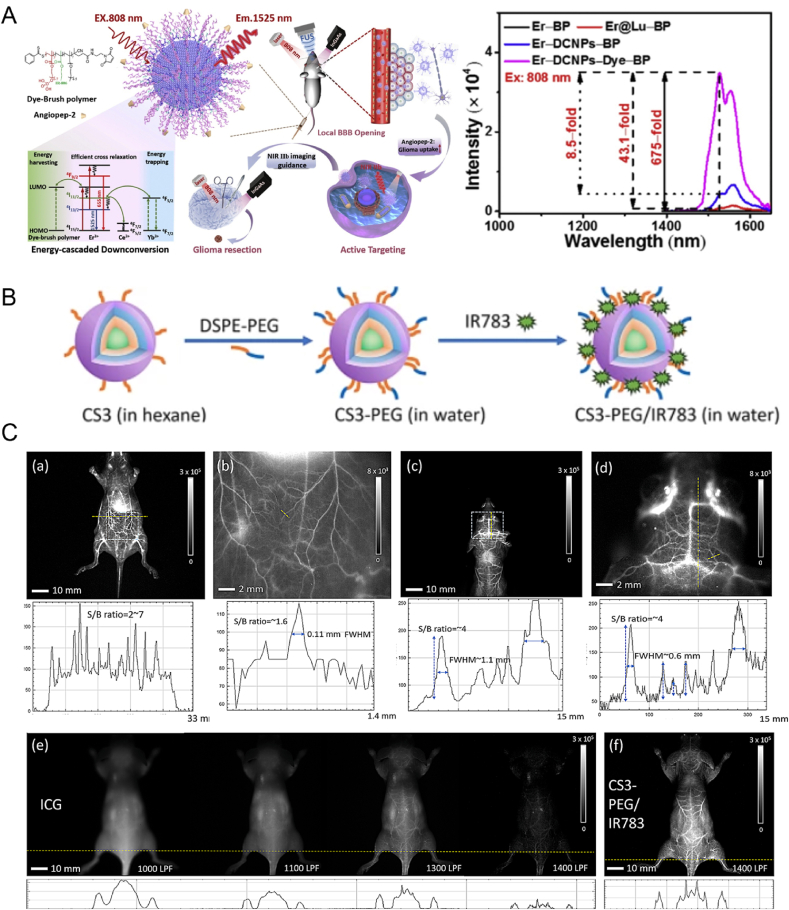
NIR-II imaging effect **A** Schematic illustration depicting the working principle of DCNP and NIR IIb emission spectra of Er-BP, Er@Lu-BP nanoparticles, Er-DCNPs-BP, and Er-DCNPs-Dye-BP [73]. **B** Schematic representation of onion-like Nd-LnNPs surface modification and dye-encapsulation process. **C** NIR-II imaging of vascular structures in a murine model [116].

Although UCL materials exhibit strong penetration under NIR excitation, the resulting emission typically falls within the ultraviolet to visible range, which is readily absorbed by biological tissues. This spectral characteristic restricts their application in deep-tissue imaging. NIR-II imaging has emerged as a rapidly advancing biological imaging technique that offers distinct advantages for *in vivo* deep-tissue imaging, including superior tissue penetration depth, enhanced spatial resolution, and reduced autofluorescence background (Fig. 11). Within the NIR-II window (1000–1700 nm), light scattering and absorption by biological tissues are substantially diminished, allowing excitation and emission light to penetrate more effectively and achieve imaging depths on the order of millimeters to centimeters. Furthermore, this spectral region exhibits minimal autofluorescence interference, leading to improved signal-to-noise ratios, imaging contrast, and spatial resolution compared with the NIR-I window. These attributes render NIR-II imaging particularly suitable for applications such as high-resolution vascular imaging, tumor boundary delineation, and dynamic monitoring of physiological processes [116,172]. To achieve efficient and bright NIR-II imaging, two primary strategies have been explored to enhance the luminescence performance of LnNPs in this spectral region: energy level modulation and dye sensitization [12,43,56,62,63,67,73,76,116,149,172,[204], [205], [206], [207]]. For instance, Ren et al. enhanced the emission intensity at 1525 nm by approximately 675-fold compared with untreated NaErF_4_ LnNPs through optimization of interlayer thickness and Ce^3+^ doping concentration [73] (Fig. 11A). The excitation spectrum can be broadened, and the intrinsic luminescence of lanthanide ions can be enhanced, through the use of broad-absorption organic dyes such as IR1061, Cy7, and indocyanine green (ICG) (Fig. 11B). In one example, Gong and colleagues reported the growth of a metal–organic framework (MOF) layer on UCNP surfaces, utilizing its porous structure to efficiently load ICG and facilitate energy transfer, thereby improving imaging performance [12]. In another study, a dye-sensitized Nd-doped nanocomposite with an onion-like structure achieved approximately 75-fold brightness enhancement in the NIR-II b region and enabled imaging at tissue depths of up to 6 mm by suppressing FL quenching and optimizing spectral matching. This system exhibited improved signal-to-background ratio and spatial resolution compared with conventional ICG, and was applied to reconstruct vascular networks at depths of 0–5 mm [116] (Fig. 11C).

#### 4.1.3 Multimodal imaging

Each individual imaging modality offers distinct advantages yet also suffers from inherent limitations. For instance, up-conversion luminescence imaging features exceptionally low background interference, but its visible-light emission restricts tissue penetration depth. Conversely, while NIR-II imaging provides superior deep-tissue resolution, it often lacks sufficient anatomical or functional context. To address these limitations, multimodal imaging strategies have been developed that integrate two or more imaging modalities to harness complementary advantages, thereby providing more comprehensive and accurate information for disease diagnosis and treatment monitoring. Leveraging LnNPs as core carriers, their tunable composition and flexible surface engineering enable the integration of multiple imaging functions into a single nanoplatform, facilitating the construction of efficient multimodal probes. A common strategy involves combining highly sensitive optical imaging with clinical imaging techniques that offer deep penetration capability. Among these, the integration of up-conversion luminescence with MRI represents a widely studied dual-modality approach. The high longitudinal relaxivity of Gd^3+^, which serves as a T_1_-weighted MRI contrast agent, is frequently employed to achieve both up-conversion luminescence and MR imaging within the same nanostructure, either by direct doping into the UCNP lattice or by incorporation into a shell layer [30,50,120,159,202]. For instance, Kostiv et al. reported core–shell NaYF_4_:Gd/Yb/Tm@NaGdF_4_ LnNPs that demonstrate this dual-modality imaging capability [202]. Building upon these developments, multimodal imaging is poised to extend beyond the mere superposition of diagnostic information toward the integration of therapeutic functions, thereby advancing the field of theranostics. In this context, Xu et al. developed a multifunctional nanoplatform that incorporates X-ray-responsive ratiometric NIR-II FL imaging with gold NP-based radiosensitization. By establishing a linear correlation between FL intensity and radiation dose, the authors implemented an adaptive radiotherapy strategy guided by real-time feedback of personalized radiation dosing. This approach resulted in reduced off-target toxicity and a 1.8-fold improvement in tumor suppression compared with conventional radiotherapy [87]. Such multimodal probes hold considerable promise for future therapeutic applications, enabling accurate localization and diagnosis while supporting interventions including photodynamic therapy, photothermal therapy, drug delivery, and gene therapy. When coupled with real-time monitoring of therapeutic efficacy, these capabilities contribute to the establishment of a closed-loop framework for diagnosis, treatment, and monitoring.

Beyond the mere integration of multiple imaging functions, surface engineering plays a critical role in mitigating potential interference between different modalities—an aspect often overlooked in simple co-doping or core-shell designs. For instance, while Gd^3+^ doping enables T_1_-weighted MRI, its high concentration or improper spatial distribution may quench UCL via energy transfer or cross-relaxation processes, especially when Gd^3+^ is placed in close proximity to emitters like Er^3+^ or Tm^3+^ [6,75]. A recent study further demonstrated that even the presence of Dy^3+^ can severely ‘poison’ up-conversion emission if magnetic and optical layers are not properly isolated, leading to complete loss of signal [208]. To address these issues, well-established surface coating strategies—such as inert shell interlayers (e.g., NaYF_4_ or NaGdF_4_ with controlled thickness)—have been shown to effectively isolate Gd^3+^ from optical reporters, preserving luminescence intensity while retaining MRI contrast performance [54,202]. For example, a 2026 study reported that a rationally designed core–shell–shell architecture (NaYbF_4_:Er@NaYbF_4_@NaGdF_4_) achieved simultaneous high-sensitivity nanothermometry and T_1_-weighted MR imaging without cross-talk between the two modalities, owing to an inner inert NaYbF_4_ layer that spatially separates the active Er^3+^ from surface Gd^3+^ [209]. Alternatively, NaLn(WO_4_)_2_ core–shell nanoplatelets have been shown to function as both T_1_/T_2_ dual-modal MRI contrast agents and NIR imaging probes, where the inert shell effectively prevents luminescence quenching by paramagnetic centers [210]. Moreover, the choice of surface functionalization—whether PEGylation, silanization, or membrane coating—must balance hydrophilicity, colloidal stability, and biocompatibility without compromising either imaging modality. For instance, GdVO_4_@YVO_4_ core–shell nanoparticles with an inert YVO_4_ intermediate layer not only prevented luminescence quenching but also maintained high r_1_ relaxivity by ensuring water proton accessibility, highlighting the importance of rational shell thickness design [211]. While excessively thick silica shells may hinder water proton accessibility to Gd^3+^, thereby reducing relaxivity, insufficient coating can lead to non-specific interactions or quenching [212]. To overcome this trade-off, advanced surface designs—including hierarchical coatings or stimuli-responsive layers—offer the possibility to dynamically modulate probe performance according to imaging needs [213,214]. A recent example is the development of core–shell–shell Gd-based nanoparticles excitable at four distinct wavelengths (272, 394, 808, and 980 nm), enabling switchable multimodal imaging under different excitation conditions without mutual interference [215]. Thus, successful multimodal imaging with lanthanide nanoparticles relies not only on compositional design but also on rational surface engineering that actively manages modality interference, ensuring each imaging channel operates optimally under physiological conditions.

### 4.2 Targeted delivery and therapeutics

Building on the achievements in high-contrast and multimodal biological imaging, the utility of LnNPs has been expanded into disease treatment, effectively bridging detection with therapeutic intervention. This includes the use of active targeting strategies, drug and gene delivery platforms, as well as photodynamic and photothermal therapies that are regulated by near-infrared light.

#### 4.2.1 Active targeting

**Fig. 12:**
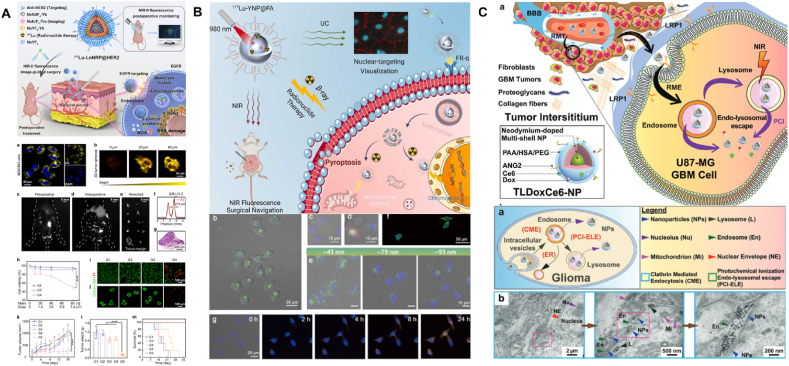
**A** Schematic Illustration Showing the Design Principle of ^177^Lu-LnNRP@HER2 Nano-Radiopharmaceutical for Postoperative Tumor Radionuclide Therapy and Prognosis Monitoring. LnNFP@HER2 and/or ^177^Lu-LnNRP@HER2 LnNRPs possess superior targeting specificity for gastric cancer cells [156]. **B** Schematic illustration showing the design strategy of tumor-cell nucleus targeting ^177^Lu-YNP@FA NPDs. The prepared Lu-YNP@FA NPD has a specific targeting ability to the nucleus of cervical cancer [153]. **C** NPs trafficking into U87-MG glioma cells and a metronomic NP regimen possessing anti-angiogenesis and directed, augmented antitumor effects. Direct observation using the TEM investigations of GBM brain sections treated with NPs [216].

Originally synthesized LnNPs rely on their intrinsic luminescence or clinically used ICG dyes for tissue illumination *in vivo*, yet they lack inherent tumor targeting capability. Consequently, precise bio-functionalization of their surface is essential for achieving active targeting. The conjugation of specific ligands onto NP surfaces enables cell-specific recognition and efficient internalization (Fig. 12A), which can enhance the accumulation of diagnostic and therapeutic agents at the lesion site while potentially reducing off-target effects. To overcome the inherent lack of targeting capability, a variety of ligands have been successfully employed for the active targeting functionalization of LnNPs (Table 10). Folic acid is widely used as a targeting moiety owing to its high affinity for folate receptors, which are overexpressed on the surfaces of various cancer cells [27,35,69,84,[153], [154], [155],^217^]. For instance, Hong et al. reported a nanoprodrug (^177^Lu-YNP@FA) designed for tumor nucleus targeting, which was constructed by functionalizing NaYF_4_:Yb/Er@NaYF_4_ core–shell nanocrystals with DSPE-PEG-FA (Fig. 12B). This prodrug was shown to specifically recognize overexpressed FRα on cervical cancer cells, undergo cellular uptake within 2 h, and achieve nuclear localization within 8 h, thereby demonstrating its targeting and nuclear delivery capabilities [153]. Such receptor-targeted NPs typically enter cells via clathrin-mediated endocytosis and are subsequently trafficked to the endolysosomal system [69,153,156]. Under acidic conditions, certain cleavable bonds incorporated into the NPs design can be disrupted, facilitating endosomal escape and enabling the release of therapeutic payloads to their intracellular sites of action. The intracellular fate of such NPs was systematically investigated by Liu et al. in a 2021 study [216] (Fig. 12C).

**Table 10:** Comparison of different active targeting strategies for LnNPs.

Targeting Strategy	Targeting Ligand/Receptor	Mechanism of Action	Internalization Pathway	Target Location	Applications	References
**Receptor Targeting**	Folic Acid (FA)	Binds to folate receptor-α (FR-α) overexpressed on cancer cells	Clathrin-mediated endocytosis	Membrane → endo/lysosome → nucleus	Used for FR-positive cancers (e.g., cervical, ovarian); enables nuclear delivery	[27,35,84,[153], [154], [155],^217^]
	Anti-HER2 antibody	Binds to HER2 receptor for specific recognition	Clathrin-mediated endocytosis	Membrane → endo/lysosome	Applied in HER2-overexpressing breast cancer; useful for post-surgical residual lesion theranostics	[156]
**Peptide Ligand Targeting**	RGD peptide (Arg-Gly-Asp)	Recognizes integrin αvβ3 on tumor neovasculature and cancer cells	Integrin-mediated endocytosis	Tumor vasculature & cell surface	Widely used for tumor imaging and therapy; good tumor penetration	[41,203,[218], [219], [220]]
**Cell-Penetrating Peptides**	TAT peptide (Trans-activating transcriptor)	Electrostatic interaction with cell membrane; direct penetration	Non-endocytic, direct translocation	Cytoplasm → nucleus	Rapid internalization; avoids lysosomal degradation; suitable for gene delivery & nuclear therapy	[46,221,222]
	Oligoarginine (e.g., R9)	Positively charged; interacts with negatively charged cell membrane	Direct penetration/membrane disturbance	Cytoplasm	Used for stem cell labeling and whole-body tracking; applicable for systemic imaging	[83]
**Organelle-Specific Targeting**	Triphenylphosphonium	Driven by mitochondrial membrane potential (ΔΨm)	Mitochondrial targeting	Mitochondria	Induces tumor ferroptosis/pyroptosis; suitable for mitochondrial damage-related therapy	[68,83,223]
	Glycyrrhetinic Acid (GA)	Binds to receptors on mitochondria of hepatocytes or certain tumor cells	Receptor-mediated endocytosis	Mitochondria	Used for hepatocellular carcinoma or specific tumor mitochondrial targeting	[113]
**Enzyme/Substrate-Responsive Targeting**	Granzyme B substrate peptide	Enzyme-cleavable release of active molecules	Endocytosis followed by enzymatic cleavage	Cytoplasm/specific organelles	Enables real-time imaging of enzyme activity *in vivo*; monitors immunotherapy response	[112]

Peptide ligands have also been explored for active targeting applications. RGD peptides, which contain the arginine-glycyl-aspartic acid sequence, represent a well-established class of targeting ligands. Integrin αvβ3, overexpressed on tumor neovascularization and on various tumor cells, can be specifically recognized by RGD peptides. Consequently, LnNPs conjugated with RGD peptides can be directed to integrin-expressing sites [41,203,[218], [219], [220]]. Beyond receptor-mediated targeting, cell-penetrating peptides have been employed to facilitate cellular internalization and nuclear delivery [46,221,222], along with other functional peptides [73,107,157,216,224,225]. In contrast to receptor-targeted strategies, cell-penetrating peptides such as TAT and polyarginine traverse the cell membrane via non-endocytic mechanisms, primarily electrostatic interactions and hydrogen bonding. This allows LnNPs to rapidly enter the cytoplasm or nucleus, thereby evading lysosomal degradation and enhancing delivery efficiency. For instance, Shi et al. conjugated TAT to DOX-loaded NaYF_4_:Yb/Er@NaGdF_4_ NPs. FL was observed at the nuclear membrane and in the cytoplasm within 3 h, with further accumulation within the nucleus observed at 6 and 24 h. The nuclear accumulation of DOX was attributed primarily to drug release from the NPs within the nucleus [221]. In addition to cellular targeting, recent studies have also extended to subcellular localization. For example, functional molecules such as triphenylphosphine and its derivatives in complex with cell penetrating peptide (CTPP) [68,83,223,226] and glycyrrhetinic acid (GA) [113] can guide LnNPs to be specifically enriched in mitochondria, respectively, providing a new way for precision medicine and biological research at the organelle level.

Despite the wide variety of targeting ligands that have been successfully conjugated to LnNPs, a critical question remains: which targeting strategy offers optimal performance? Direct head-to-head comparisons under identical experimental conditions are scarce in the literature, yet the available studies provide valuable insights (Table 11). Currently, the targeting ligands conjugated onto LnNPs can be broadly classified into three functional categories: membrane receptor-targeting ligands (e.g., folic acid, RGD peptides, and antibodies), organelle-targeting ligands (e.g., triphenylphosphonium and glycyrrhetinic acid), and cell-penetrating peptides (e.g., TAT and oligoarginine).

**Table 11:** Comparison of different types of targeted effects in existing literature.

Targeting Strategy	Ingestion *in vitro*	Accumulation *in vivo*	Subcellular precision	Reference
**Receptor Targeting**	++++	++++	++	[153,156,227]
**Peptide Ligand Targeting**	+++++	+++	+++++	[41,203,218,220,221]
**Cell-Penetrating Peptides**	+++++	+	+++++	[46,228]
**Organelle-Specific Targeting**	++++	+	+++++	[68,113,223,228]
**Dual/Multi-Ligand Targeting**	+++++	++++	++++	[68,229]

For receptor-targeting ligands, Cinar et al. directly compared FA and RGDK-functionalized NaYF_4_:Yb/Er UCNPs in MCF-7 breast cancer cells [227]. Both ligands, conjugated via PAA coating, exhibited remarkably higher cellular uptake compared to non-targeted controls, with comparable targeting efficiency between FA and RGDK. This suggests that the choice between these two small-molecule ligands may depend more on the receptor expression profile of the target cells rather than intrinsic ligand superiority. By comparison, Liu et al. compared TAT peptide-functionalized NaYF_4_:Yb/Er@NaGdF_4_ NPs with non-targeted controls in HeLa cells [221], demonstrating that TAT conjugation enabled nuclear membrane and intranuclear accumulation within 3–6 h, a delivery efficiency superior to receptor-mediated endocytosis which typically localizes in endo/lysosomal compartments. However, it is worth noting that this faster and more direct cytoplasmic/nuclear delivery comes at the cost of lower cell type specificity.

In addition, a particularly instructive comparison was reported by Aubrun Fulbert et al., who evaluated PEG-versus tripolyphosphate (TPP)-coated LaF_3_:Ce LnNPs in pancreatic tumor models [228]. Although TPP-coated NPs exhibited markedly higher accumulation in tumor spheroids *in vitro* (11.5-fold greater than that of PEG-coated NPs, as measured by XRF microtomography) and a higher dose enhancement factor (DEF = 2.08 vs. 1.73 for PEG-coated NPs in PANC-1 spheroids), their *in vivo* performance was considerably inferior. Specifically, TPP-coated NPs were rapidly cleared from the bloodstream (<1% ID/g at 5 min post-injection, compared with 25% ID/g for PEG-coated NPs), resulting in approximately 2.3-fold lower tumor accumulation at 4 h and 10-fold lower at 24 h, alongside extensive non-specific uptake in the liver and spleen (>90% ID/g in the spleen at 1 h). This stark contrast underscores that, beyond receptor–ligand affinity, surface charge, colloidal stability, and circulation half-life are equally critical determinants of overall targeting efficiency *in vivo*.

When comparing different targeting strategies across studies, several trends emerge: (1) receptor-targeting ligands (FA, RGD, anti-HER2) offer cell-type specificity but typically rely on clathrin-mediated endocytosis, leading to endo/lysosomal entrapment. (2) cell-penetrating peptides (TAT, oligoarginine) achieve superior cytoplasmic/nuclear delivery via direct membrane translocation, but lack cell specificity. (3) organelle-targeting moieties (CTPP, GA) enable precise subcellular localization but may introduce positive surface charge that compromises *in vivo* circulation. Collectively, the optimal targeting strategy is highly context-dependent: for *in vitro* cell imaging, FA and RGD offer facile conjugation and reliable enhancement; for rapid cytoplasmic/nuclear delivery, CPPs are preferred; for subcellular precision, TPP is superior *in vitro* but requires PEGylation or other shielding strategies for *in vivo* application.

However, it should be noted that these functional categories are not mutually exclusive. In practice, researchers often co-conjugate two or more ligands onto the same LnNPs to synergistically combine their strengths. For example, Wang et al. compared the cellular uptake of dual-targeting UCNPs-C_60_ versus single-targeting APBA-UCNPs or HAC_60_ in PC12 cancer cells [230]. The dual-targeting probe exhibited significantly higher intracellular accumulation, with approximately twofold greater mean FL intensity. Blockade of PSA and CD44 receptors reduced uptake by 34.8% and 31.6%, respectively, suggesting cooperative function of dual receptor-mediated endocytic pathways. In parallel with the above dual-functionalization strategy, researchers have extended ligands to peptide-peptide combinations that simultaneously recognize different receptors overexpressed on tumor cells or tumor blood vessels. Cao et al. demonstrated the endocytic behavior of A549 cells toward polydopamine-coated up-conversion nanoparticles (UCNP@PDA) and the dual-targeting peptide (RGD10-NGR9) modified nanoprobe (UCNP@P-RGD-NGR) using ICP-MS quantification and cellular imaging [229]. The results showed that, under identical incubation conditions, the dual-targeting modification significantly increased the cellular uptake of Y^3+^ compared with the non-targeting control, with an approximately 11-fold increase at a concentration of 160 μmol/L. This further indicates that the synergistic action of the two ligands can greatly enhance the binding affinity of the LnNPs to the tumor cell surface. Subsequent competitive binding assays also confirmed that excess free RGD10-NGR9 peptides could effectively block the binding of the nanoprobe to cells, thereby verifying that the uptake process is mediated by specific recognition between the surface peptides and their target receptors (integrin αvβ3/αvβ5 and aminopeptidase N). Regarding receptor targeting, FA remains the most common ligand. Notably, beyond conventional dual-ligand uptake enhancement, recent studies have explored functional combinations to amplify efficacy. For example, Zhu et al. co-modified UCNPs with FA and croconaine (UCNP-Cro/FA), where FA ensures tumor specificity and croconaine promotes lysosomal iron deposition, thereby enhancing ferroptosis/pyroptosis via lysosomal membrane permeabilization [69].

Currently, representative examples of ligand multiplexing have been extended to dual-targeting peptides for gene delivery [231], exosome-based natural multi-ligand platforms [232], and indirect ligand multiplexing strategies [68,233]. In many cases, a dual or multi-ligand design, rather than a single ligand, provides the best balance of specificity, penetration, and subcellular accuracy, and should be tailored to the specific biological barrier and therapeutic goal.

#### 4.2.2 Drug delivery, gene therapy and controlled release

**Fig. 13:**
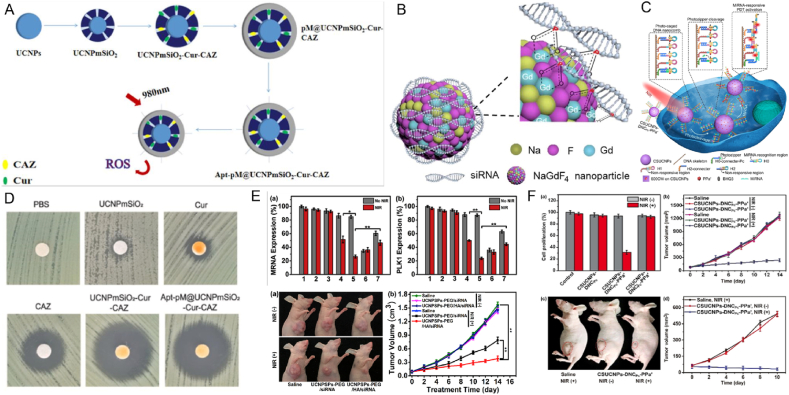
**A** The preparation process of Apt-pM@UCNPmSiO_2_-Cur-CAZ and **(D)** In vitro antibacterial effect of Apt-pM@UCNPmSiO_2_-Cur-CAZ [185]. **B** Schematic diagram of the interaction between siRNA and the surface of LnNPs [49]. **C** Illustration of NIR photo-switched miRNA amplifier for precise PDT and **(F)** Proliferation of HepG2 cells treated with CSUCNPs-DNCPc,CSUCNPs-DNC’Pc -PPa’, and CSUCNPs-DNCPc -PPa’ before and after808-nm light [234]. **E** Gene silencing and cell proliferation inhibition. Expression levels of PLK1 mRNA detected with RT-qPCR and PLK1 protein detected with ELISA, and *In vivo* antitumor therapy [100].

Following successful active targeting of LnNPs to diseased cells, the efficient delivery of therapeutic drugs or biomolecules to the target site, along with controlled and on-demand release, represents a critical factor influencing therapeutic outcomes. A range of loading strategies based on LnNPs have been developed to address diverse therapeutic requirements. These include electrostatic adsorption, covalent conjugation [49,51,95], loading within mesoporous silica [92,94,97], and core–sandwich assembly [43,90]. For instance, Liu et al. constructed a NaYF_4_:Yb/Er@mSiO_2_ nanoplatform loaded with doxorubicin (DOX). This system utilizes UCL for tumor FL imaging, and the DOX loaded within the mesoporous silica enables real-time monitoring of drug release and therapeutic efficacy under 980 nm laser irradiation [94]. This strategy has subsequently been extended to multi-drug co-loading applications. For instance, Nie and colleagues reported the use of the mesoporous structure of UCNPs@mSiO_2_ to co-load the photosensitizer curcumin (Cur) and the antibiotic ceftazidime (CAZ). This system was shown to exhibit enhanced antibacterial efficacy through a synergistic mechanism combining chemical antibacterial activity with photodynamic antibacterial effects [185]. This synergistic delivery platform acts on bacteria via multiple pathways. Ceftazidime provides direct antibacterial activity, while curcumin contributes photodynamic antibacterial effects along with anti-biofilm, anti-inflammatory, and tissue repair-promoting properties (Fig. 13A–D). Concurrently, the development of precise delivery systems based on LnNPs has extended therapeutic strategies to target the genetic basis of diseases. Gene therapy represents a pivotal strategy in precision medicine that facilitates the delivery of functional nucleic acids, including siRNA (Fig. 13B), miRNA (Fig. 13C), plasmid DNA, and CRISPR-Cas9 components, thereby enabling precise regulation of disease-associated gene expression at the transcriptional or translational level for therapeutic purposes. [46,49,159,234]. For instance, Zhang and colleagues developed a near-infrared light-responsive microRNA amplifier for precision photodynamic therapy in early-stage cancers, utilizing photo-caged DNA nanocombs and UCNPs [234]. In this system, a cleavable optical zipper within the amplifier blocks the miRNA recognition region, thereby preventing off-target effects induced by circulating miRNAs. Upon near-infrared irradiation, ultraviolet emission from the UCNPs enables precise photocleavage of the optical zipper from the designed photo-caged DNA nanocomb. This exposes the miRNA recognition region, initiating a cascade that activates all photosensitizer molecules within the amplifier. Subsequently, under blue light emission from the UCNPs, substantial ROS are generated to inhibit tumor proliferation and induce apoptosis (Fig. 13F). Such approaches offered the potential for intervention at the origin of disease processes. However, nucleic acid molecules face several challenges that limit their clinical application. These include poor cell membrane permeability, low stability *in vivo*, susceptibility to enzymatic degradation, and potential off-target effects. Several strategies have been explored to address these challenges. One approach involves the electrostatic adsorption of negatively charged nucleic acids onto positively charged LnNPs. The generation of such positive surface charge is attainable via either removal of surface ligands to expose Ln^3+^ ions or functionalization with cationic polymers, including PEI and poly-L-lysine (PLL). This approach enables the assembly of nanocomposites, as reported by Yu et al. [49]. However, this approach may be associated with reduced delivery efficiency and an increased risk of off-target effects. Another strategy focuses on achieving precise control over release kinetics through encapsulation approaches that enable stimulus-responsive release systems. Such systems can facilitate controlled release of nucleic acids within specific organelles or at target sites in response to stimuli such as pH, enzymatic activity, or light [226,235]. A variety of stimulus-responsive release systems have been integrated into LnNPs, including those triggered by light [43,87,95], pH [49,51], enzymatic hydrolysis [95,107,112,157], and redox reactions [56]. Among these, light-triggered release has received considerable attention owing to its spatiotemporal controllability and non-invasive nature. For instance, Ju and colleagues developed photocleavable up-conversion nanocapsules for spatiotemporally controlled gene delivery [100]. In this system, siRNA is encapsulated within the carrier and remains stably sequestered in the absence of near-infrared light, thereby preventing premature leakage and non-specific release during circulation. Upon exposure to near-infrared light at the target site, the UCNP core converts long-wavelength excitation into ultraviolet emission, which cleaves the photocleavable linker connecting the siRNA, enabling on-demand, rapid, and localized release. This system was reported to silence over 80% of target genes, achieve approximately 70% tumor growth inhibition in an animal model, and improve survival rates in the treated group (Fig. 13E). Beyond single-mode light-controlled release, strategies combining light responsiveness with endogenous tumor microenvironment stimuli have been explored to achieve spatiotemporal delivery with enhanced precision. Chen et al. recently demonstrated the effectiveness of such a collaborative approach [159]. Their platform was capable of co-delivering distinct nucleic acid cargos with different functions, including Cas9 ribonucleoprotein for gene editing and a DNAzyme system for gene silencing, thereby achieving synergistic regulation of two immune checkpoint genes, Ptpn2 and PD-L1. Additionally, the conjugation of peptide substrates or their recognition units to LnNP surfaces for the construction of enzyme-responsive therapeutic platforms offers methodological insights for developing analogous cleavable linker-based nucleic acid delivery systems [107,157].

Notably, intelligent platforms that integrate diagnostic and therapeutic functions with multiple controlled release capabilities are emerging. In particular, Chen and colleagues reported an integrated theranostic platform based on NaNdF_4_ LnNPs that exhibit both photothermal conversion and NIR-II luminescence properties [95]. Among the emerging carriers, metal–organic frameworks (MOFs) have gained attention as intelligent nanozymes with dual catalytic and theranostic functions [236]. Their high porosity, tunable pore size, and enzyme-mimetic activities make them attractive for co-delivery of drugs, nucleic acids, and photosensitizers [12,128]. When combined with LnNPs, MOF coatings can provide not only high loading capacity but also catalytic therapeutic effects (e.g., chemodynamic therapy) that complement phototherapy, further enriching the functionality of LnNP-based theranostic systems.

#### 4.2.3 Photodynamic/photothermal therapy

LnNPs have been extensively investigated for phototherapy applications, including photodynamic therapy (PDT) and photothermal therapy (PTT), owing to their unique optical properties and photothermal conversion capabilities [12,21,68,85,90,92,93,95,113,127,128,177,179,180,207,223,225,226,[237], [238], [239], [240], [241], [242], [243]]. In PDT, photosensitizer molecules generate ROS, such as singlet oxygen (^1^O_2_), which could induce cytotoxicity in adjacent cancer cells. Conventional PDT typically relies on visible or ultraviolet light excitation, which suffers from limited tissue penetration depth and potential phototoxicity. UCNPs can convert near-infrared excitation (e.g., 980 nm) into shorter-wavelength emission, thereby activating loaded photosensitizers and enabling PDT in deep tissues [239].

**Fig. 14:**
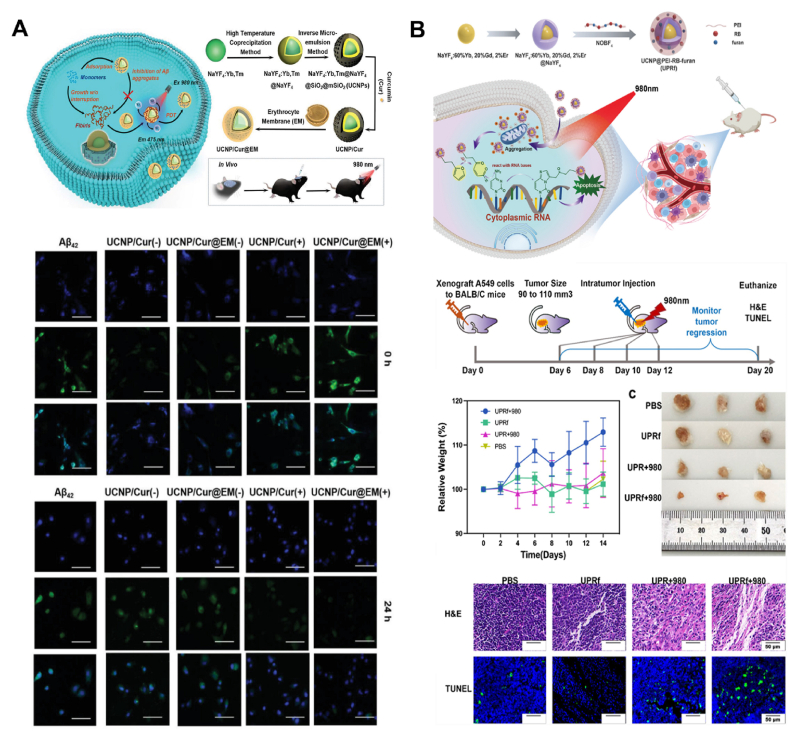
A Schematic illustration of biomimetic up-conversion nanobait-based PDT for the inhibition of Aβ aggregates [179]. Intracellular ROS in SK-N-SH cells indicated by DCFH-DA after incubation with UCNP/Cur or UCNP/Cur@EM with (+) or without (−) NIR illumination. **B** Schematic illustrations of a synthetic route of UCNP@PEI-RB-furan (UPRf) b furan-mediated RNA target and NIR modulated photodaynamic cancer therapy [237]. Scheme of workflow for A549 xenograft tumor model and the following *in vivo* treatment procedures.

The current strategy for constructing up-conversion PDT systems involves doping rare earth ions (such as Tm^3+^ and Er^3+^) into the UCNP lattice. Upon near-infrared excitation, these ions emit ultraviolet or visible light, which subsequently activates photosensitizers to generate ROS via energy transfer to molecular oxygen [93,179,226,238]. This approach offers several advantages, including structural simplicity, compositional stability, and a direct energy transfer pathway, thereby potentially circumventing issues associated with exogenous photosensitizers, such as leakage or photobleaching. Consequently, these features render this strategy particularly suitable for *in vivo* applications where long-term material stability is desired. For example, Wang et al. reported an interesting study (Fig. 14A) in which intracellular ROS increased in SK-N-SH cells when cultured with free Aβ peptide, indicating that SK-N-SH cells suffered from oxidative stress caused by Aβ aggregates. After 24 h of incubation, the FL intensity was further enhanced, indicating an enhanced oxidative stress response under continuous stimulation with Aβ peptide. UCNP (NaYF_4_:Yb/Tm@NaYF_4_@SiO_2_@mSiO_2_)/Cur or UCNP/Cur@EM without NIR irradiation were initially able to inhibit ROS production, but ROS were clearly detected after 24 h of incubation, showing limited inhibition efficiency in the absence of PDT. The antioxidation of Cur can temporarily reduce ROS, but the remaining Aβ aggregates trigger A sustained oxidative stress effect, leading to elevated ROS after 24 h. In contrast, temporarily too high ROS is generated by PDT effects of illuminated UCNP/Cur or UCNP/Cur@EM, which can be used to target Aβ photooxygenation and dissolve pre-existing Aβ aggregates, thereby reducing the toxic stimulus of oxidative stress. As expected, weak FL was found in cells treated with illuminated UCNP/Cur and UCNP/Cur@EM after 24 h, which can be attributed to A temporary increase in PDT effect that disappeared after 24 h when Aβ peptide was treated with UCNP/Cur or UCNP/Cur@EM under near-infrared irradiation. This indicates that oxidative stress levels *in vitro* are reduced by inhibition of Aβ aggregates [179].

However, the efficacy of PDT depends not only on the luminescence intensity of the NPs but also on their accumulation efficiency and singlet oxygen generation rate within tumor cells. Therefore, an alternative strategy involves loading or conjugating exogenous photosensitizers onto UCNP surfaces, including organic photosensitizers such as Ce6, RB, and ZnPc, as well as inorganic photosensitizers like TiO_2_, through physical adsorption, covalent coupling [12,92,113,127,207,223,237], or encapsulation [126]. This approach allows for the selection of photosensitizers with high singlet oxygen quantum yields or broad absorption spectra, enabling the construction of excitation-emission activation cascades through interface engineering. In this context, Cheng and colleagues proposed a cancer treatment strategy [237] that achieves targeted photodamage to cytoplasmic RNA and mitochondria with precise activation controlled by UCNPs. In this system, PEI-modified furan molecules are covalently cross-linked with the photosensitizer RB and subsequently coated onto UCNPs composed of NaYF_4_: 60% Yb/20% Gd/2% Er@NaYF_4_. The emission of these UCNPs in the 530 to 550 nm range provides an optimal signal for activating the photosensitizer RB, leading to the generation of reactive ROS, including ^1^O_2_, upon exposure to 980 nm NIR light. The generated ^1^O_2_ induces a ring-opening reaction of the furan moiety, enabling its covalent anchoring to RNA. The close proximity of UCNP@PEI-RB-furan to RNA facilitates efficient RNA damage by the ROS produced from RB. Degradation of the targeted RNA results in cellular dysfunction and subsequent cell death (Fig. 14B).

In practice, these two strategies are often combined to leverage their respective strengths, and in some cases, integrated with gene therapy to achieve synergistic treatment outcomes. For instance, Liu et al. recently reported a core–shell hybrid nanosystem designed to enhance radio-photodynamic therapy [207]. This system consisted of Eu^3+^-doped NaGdF_4_ nanocrystals coated with an aggregation-induced emission (AIE) photosensitizer (TQ) and an amphiphilic polymer (DSPE-PEG_2000_). The nanocrystals efficiently absorbed high-energy radiation from the decay of the radionuclide ^18^F and directly sensitized TQ through triplet energy transfer. This approach circumvented inefficient energy transfer pathways, achieving an energy transfer efficiency of approximately 100% and enhancing singlet oxygen production. In a 4T1 tumor-bearing mouse model, the ^18^F-stimulated nanosystem exhibited sustained NIR-II FL and efficient ROS generation at the tumor site relative to control groups. Tumor growth was inhibited with no apparent systemic toxicity. Song and colleagues constructed a UCNP-based optogenetic nanosystem for constructing PDT and treating malignancies via cascade gene therapy [226]. NaYF_4_@NaYF_4_:Yb/Tm@NaYF_4_ UCNPs are directed to mitochondria by covalently modifying TPP and subsequently activated *in situ* by the LRET process under NIR laser excitation of genetically encoded photosensitizers. Meanwhile, siRNA specific for Bcl-2 mRNA was covalently modified to the surface of UCNP via ROS-sensitive bonds. Thus, photogenerated ROS would subsequently stimulate siRNA release to perform gene therapy in a controlled manner. This optogenetic tool induced a high apoptosis rate (60%) and a marked inhibition of tumor volume.

PTT is a non-invasive therapeutic modality that employs photothermal conversion agents to generate localized hyperthermia under near-infrared light irradiation, thereby ablating tumor tissue. Although LnNPs themselves are not conventional photothermal agents, they can be endowed with photothermal conversion properties through strategies such as constructing core–shell structures or modulating ion doping. For instance, photothermal conversion can be enhanced by combining LnNPs with materials that exhibit pronounced photothermal responses, such as gold nanoshells or copper sulfide, or by introducing ions with near-infrared absorption, such as Nd^3+^ or Yb^3+^ [90,177,180,225,[241], [242], [243]]. In recent years, combining PDT with PTT or further integrating other treatment modalities, including chemotherapy, immunotherapy, and radiotherapy, has emerged as a strategy to enhance cancer treatment efficacy [226,[244], [245], [246]]. Beyond conventional combination partners, emerging two-dimensional materials such as MXenes have recently been explored as versatile photothermal and theranostic platforms. Surface-engineered MXene–polymer–metal hybrids exhibit strong near-infrared absorption, high photothermal conversion efficiency, and facile multifunctionalization, offering promising opportunities for integration with LnNPs to achieve synergistic photothermal/photodynamic therapy and multimodal imaging [247].

### 4.3 Integration of diagnosis and treatment

With the deep integration of nanotechnology and biomedicine, the unique optical imaging and excellent biotherapeutic functions of LnNPs are allowed to be integrated into the same nano platform, which gradually promotes the transformation from the traditional mode of separation of diagnosis and treatment to the integration of diagnosis and treatment. At present, a variety of integration strategies, including core-shell structure integration, surface engineering cooperation, and stimulus-responsive intelligent platform construction, have been greatly developed. Among them, core-shell structure integration is one of the most classical strategies. By constructing core-shell or sandwich structure, the imaging unit and treatment unit are packaged at different levels to achieve physical isolation and synergy of functional modules. For example, UCL and MRI dual-modality guided chemotherapy system, and NIR II imaging-guided photothermal chemotherapy synergistic therapy and immunotherapy platform have shown good application prospects [85,94,117,202,248]. Different from the idea of physical separation of core-shell structure, surface engineering collaborative strategy focuses on chemical modification and functional assembly on the surface of NPs. For example, early studies achieved tumor targeting, FL imaging and chemotherapy by co-modifying folic acid and doxorubicin on the surface of UCNPs [118]. In recent years, this strategy has been further extended to the field of radiodiagnosis and treatment, such as the construction of a nano-platform for FL imaging-guided targeted radiotherapy by surface modification of targeting units and radionuclide chelators [153], or the diagnosis and treatment of postoperative residual lesions with the function of single photon emission computed tomography (SPECT) and internal irradiation [156]. The specific construction and application of the stimulus response platform have been comprehensively summarized by previous teams, and the relevant information can be found in their manuscripts [235].

## 5 Pharmacokinetics and biodistribution

Pharmacokinetics and biodistribution are crucial parameters in evaluating the biological behavior of nanomaterials following administration. Pharmacokinetics describes the absorption, distribution, metabolism, and excretion (ADME) of NPs, while biodistribution refers to their localization and accumulation in various tissues and organs over time. Understanding these processes is essential for assessing the therapeutic potential, safety profile, and clearance mechanisms of LnNPs. Critically, the surface engineering strategy applied to LnNPs is the primary determinant of their *in vivo* fate, governing not only their colloidal stability in circulation but also their clearance route, tissue accumulation patterns, and long-term toxicity. From a macroscopic perspective, the diverse hydrophilic modification approaches can be categorized into three distinct classes based on their impact on pharmacokinetics: (1) non-PEGylated surfaces (including ligand-free bare LnNPs and ligand-oxidized surfaces), (2) PEGylated surfaces (achieved via ligand exchange or polymer encapsulation), and (3) inorganic SiO_2_ surfaces. These categories exhibit fundamentally different pharmacokinetic behaviors, which we systematically compare below.

### 5.1 Non-PEGylated surfaces LnNPs

LnNPs that lack a protective PEG or inert shell exhibit the poorest pharmacokinetic profiles. Their common feature is the partial or complete exposure of the inorganic surface, which leads to rapid and unfavorable biological interactions.

Ligand-free LnNPs, prepared by acid treatment or NOBF_4_-mediated ligand stripping, possess exposed Ln^3+^ ions on their surface. These bare metal sites rapidly coordinate with endogenous phosphates, carbonates, and plasma proteins upon intravenous administration, leading to instantaneous aggregation and opsonization. Consequently, these particles are cleared within minutes by the mononuclear phagocyte system (RES), with >90% of the injected dose accumulating in the liver and spleen [43,47,49]. Moreover, this rapid clearance is accompanied by significant retention. Studies have shown that ligand-free particles exhibit negligible excretion over extended periods, with detectable residuals persisting in the liver for months. The exposed Ln^3+^ ions also pose a substantial toxicity risk, as they can leach from the NPs surface and interact with biological macromolecules, potentially contributing to off-target effects and long-term tissue damage. Similarly, ligand-oxidized LnNPs offer only modest improvement [[16], [17], [18],^21^]. While the generated carboxylate groups confer initial water dispersibility, the oxidized layer is prone to hydrolysis and decarboxylation under physiological pH, and these particles lack steric stabilization. They suffer from extensive protein corona formation and rapid RES uptake, with significant aggregation observed within 24 h in complete serum-containing media. The lack of a protective, non-fouling layer renders both ligand-free and ligand-oxidized LnNPs unsuitable for long-circulating *in vivo* applications, as their rapid clearance, poor excretion, and potential toxicity present major barriers to clinical translation. This class of surface modification, while useful for *in vitro* assays or as an intermediate step for further functionalization, does not meet the requirements for effective *in vivo* theranostics.

### 5.2 PEGylated surfaces LnNPs

In stark contrast to non-PEGylated counterparts, the introduction of PEG chains—whether via ligand exchange with PEGylated ligands (e.g., PEG-phosphate, PEG-dicarboxylic acid, DSPE-PEG) or through polymer encapsulation with amphiphilic block copolymers—substantially improves the pharmacokinetic profile of LnNPs. PEGylation serves a dual purpose: it shields the NPs surface from non-specific protein adsorption, thereby reducing opsonization and delaying RES recognition, and it provides a hydrophilic, sterically stabilizing layer that maintains colloidal stability in complex biological fluids.

As a result, PEGylated LnNPs exhibit significantly prolonged blood circulation times compared to non-PEGylated formulations. For example, Cheng and colleagues reported that PEG-coated NaYF_4_ LnNPs maintained detectable blood concentrations for several hours post-injection, with a circulation half-life substantially longer than that of bare particles [249]. The biodistribution pattern remains predominantly hepatic and splenic, but the extent of accumulation is markedly reduced. Li and colleagues further corroborated this observation using laser ablation inductively coupled plasma mass spectrometry (LA-ICP-MS) [250], revealing that PEGylated NaYF_4_:Yb/Tm/Gd LnNPs distributed primarily to the liver and spleen, with significant but lower levels detected in the kidneys, heart, and lungs. At the sub-organ level, PEG-UCNPs mainly accumulated within the red pulp of the spleen rather than the white pulp, indicating that these LnNPs are poorly immunogenic, or not immunogenic at all. The primary clearance organ for PEG-UCNPs was the liver, although they accumulated more prominently in the spleen. Importantly, LA-ICP-MS imaging of Fe, Cu, and Zn in the kidney concluded that PEG-UCNPs do not exhibit nephrotoxicity.

Critically, unlike ligand-free particles, PEGylated LnNPs are progressively cleared over time, with clearance efficiency being influenced to some extent by particle size. For instance, Machová Urdzíková et al. reported that PEG-coated large-sized UCNPs (L-UCNP@Ale-PEG) were primarily eliminated via the hepatobiliary route and could be almost completely cleared from the liver within 96 h post-injection [193]. Aubrun Fulbert et al. compared PEG- (∼45 nm) and TPP- (∼257 nm) coated LaF_3_:Ce NPs, both of which predominantly accumulated in the liver and spleen. PEG-coated NPs were progressively cleared from organs, with lanthanum concentrations in all organs at 14 days post-injection being lower than those at 24 h. In contrast, TPP-coated NPs exhibited irreversible accumulation in the liver and spleen (>90% ID/g at 1 h), with negligible clearance over time [228]. A substantial body of research supports the currently accepted size-dependent clearance paradigm: NPs smaller than 10 nm undergo renal clearance, whereas NPs larger than several tens of nanometers are cleared via the hepatobiliary route [[251], [252], [253]].

As discussed in multiple reviews, PEG-coated formulations typically exhibit higher solubility, longer cycle times, and reduced immunogenicity and antigenicity, as confirmed by numerous studies [254,255].

### 5.3 Inorganic SiO_2_ surface LnNPs

Among all surface engineering strategies, coating LnNPs with a SiO_2_ shell, whether amorphous or mesoporous, provides the most robust pharmacokinetic profile. The SiO_2_ layer forms an inert, chemically stable, and biocompatible interface that effectively isolates the inorganic core from the biological environment, preventing both ion leaching and non-specific protein adsorption.

The biodistribution of SiO_2_-coated LnNPs generally mirrors that of PEGylated counterparts, with predominant accumulation in the liver and spleen [105,256]. However, the key distinction lies in the superior long-term colloidal stability and the remarkably low toxicity profile of SiO_2_-coated particles. More recently, Zhou et al. systematically investigated the bioavailability, biodistribution, and toxicity of silica-coated UCNPs administrated by gavage [257]. Their results demonstrated that these LnNPs can permeate the intestinal barrier and enter blood circulation, as confirmed by microstructure observation of Peyer's patches in the intestine. Specifically, NaYF_4_:Yb/Er@SiO_2_ LnNPs mainly accumulate in bone, stomach, and intestine by oral administration, while these LnNPs mainly accumulate in liver and spleen by intravenous administration. Most importantly, even after 14 consecutive days of oral administration at a high dose of 100 mg/kg, NaYF_4_:Yb/Er@SiO_2_ in mice showed no significant toxicity.

However, tissue-level bulk accumulation data are insufficient to reveal the true localization of LnNPs within the microenvironment. Using elemental imaging techniques, Kostiv et al. precisely mapped the distribution of intravenously administered SiO_2_-coated UCNPs down to the vascular scale, thereby filling a gap in previous studies regarding intravascular retention and the influence of organ barriers [258]. They performed LA-ICP-MS elemental imaging on tissue sections harvested 24 h post-injection from B16F10 melanoma-bearing mice. The results showed that in tumor tissues, the Gd signal (26 μg/g) clearly delineated the vascular contours with a sensitivity far superior to that of the endogenous Fe signal (410 μg/g, derived from hemoglobin), indicating that the particles predominantly remained within the tumor neovasculature rather than extravasating into the interstitium. Notably, in healthy tissues, the Gd signal revealed selective localization. In the liver, the Gd signal was not only confined to the vasculature but also extended to the perivascular spaces, with high accumulation occurring in both vascular and perivascular regions, consistent with Kupffer cell uptake. This hepatic retention aligns with the known role of the liver in particle clearance. In the brain, however, due to the intact blood–brain barrier, the particles were strictly limited to the vascular lumen and did not penetrate into the brain parenchyma. These distribution features were in good agreement with immunohistochemical staining for endothelial cells (CD31). These results suggest that differences in vascular permeability may play a decisive role in the distribution pattern.

Given that non-degradable SiO_2_-coated LnNPs tend to accumulate persistently in the liver and spleen (as discussed above), degradable silica shells have recently attracted interest as a means to facilitate post-treatment clearance. In this context, a study on degradable silica-shell-based probes (NaNdF_4_@DMS-Aly) reported that after therapy [95], these nanohybrids were predominantly translocated from the lungs to the liver and spleen, with the signals gradually diminishing after 48 h and being largely cleared by day 8. This indicates that, similar to the non-degradable counterparts, the RES serves as their primary clearance pathway. Importantly, however, when recovered from the liver, the particle size was found to have decreased from the original 220 nm to approximately 38 nm, suggesting that these smaller fragments are more readily excreted via the renal or hepatobiliary system. Such degradation-induced size reduction offers a potential advantage over non-degradable silica coatings, which may linger in the RES for extended periods.

### 5.4 Comparative summary and strategic guidance

In fact, many recent reviews or research articles have been published on understanding how different delivery routes affect the *in vivo* fate of UCNPs [252,253,259,260]. For example, a recent systematic review by Khan et al. comprehensively evaluated the toxicity, biodistribution, and biosafety of UCNPs based on studies published from 2008 to 2024, covering key toxicological pathways including oxidative stress, ROS generation, inflammatory responses, and apoptosis, as well as the ADME processes. Based on the above analysis, we systematically compare the biodistribution and clearance profiles of LnNPs with different surface Bioengineering (Table 12).

**Table 12:** Comparative summary of in vivo biodistribution, circulation, and clearance profiles of surface-engineered LnNPs.

Strategies	Clearance Route	Clearance Efficiency/Half-Life	Key Features	References
**TPP Coating/Small Ligand Coating**	No effective clearance	Very poor; irreversible accumulation in liver/spleen (>90% ID/g)	Poor *in vivo* safety	[228]
**Exposed Ln^3+^**	No effective clearance	Instantaneous aggregation	Severe ion leaching and toxicity risks	[43,47,49]
**PEGylation (<10 nm)**	Renal (primary)/Hepatobiliary	Fast; partial excretion via urine	Favorable rapid clearance	[23]
**PEGylation (>10 nm)**	Hepatobiliary (primary)	Moderate; progressive clearance over time	Less efficient clearance than ultra-small counterparts	[249]
**PEGylation (Self-Consuming Shell)**	Hepatobiliary	Long circulation (∼74 min); >90% reduction in protein corona	Extended circulation; breakthrough anti-protein corona strategy	[261]
**Lipid Encapsulation**	Hepatobiliary (primary, ∼90%)	Fast; liver t_1/2_ = 23.0 h, spleen t_1/2_ = 14.9 h	Engineered clearance with high efficiency	[262]
**PAA Coating**	Hepatobiliary (extremely slow)	Extremely slow; detectable residual at 115 days	Long-term retention; poor clearance	[66]
**SiO_2_ Coating (Amorphous)**	Hepatobiliary	Moderate	No overt toxicity observed at high dose (100 mg/kg)	[256]
**SiO_2_ Coating (Degradable)**	Hepatobiliary → Renal (after degradation)	Enhanced than Amorphous	Degradable design reduces long-term retention; Fragments more readily excreted	[95]

## 6 Toxicity assessment of LnNPs

**Fig. 15:**
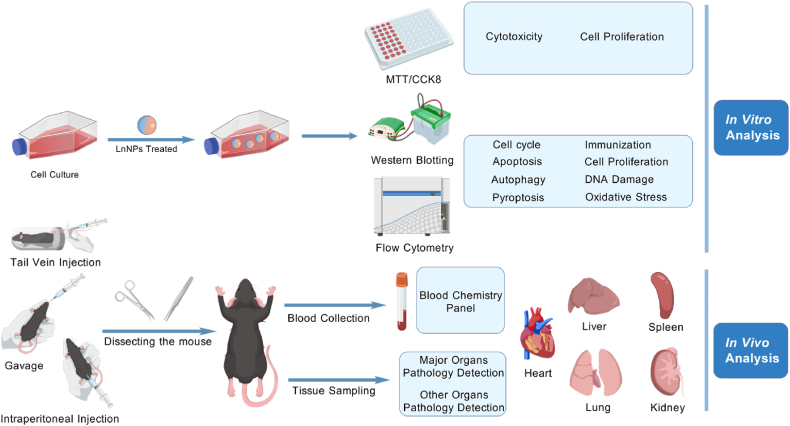
Schematic depiction of the detectable indicators for LnNPs toxicity in preclinical settings, using *in vitro* and *in vivo* models.

The successful clinical translation of LnNPs hinges not only on their optical performance and therapeutic efficacy but also on a comprehensive understanding of their safety profile. While the preceding section has detailed the pharmacokinetics and biodistribution of surface-engineered LnNPs, a systematic evaluation of their cytotoxicity and *in vivo* toxicity is equally critical (Fig. 15). This section systematically compiles and analyzes the available *in vitro* cytotoxicity data (e.g., IC_50_/CC_50_ values) and *in vivo* maximum tolerated dose (MTD) data for various surface-modified LnNPs across different cell lines and animal models. Importantly, these data are contextualized by the surface engineering strategy employed, as surface chemistry is the primary determinant of the biological interactions and toxicity profiles of LnNPs.

### 6.1 Non-PEGylated surfaces LnNPs

LnNPs lacking a protective PEG or inert shell generally exhibit the poorest safety profiles, primarily due to exposed Ln^3+^ ions that promote protein adsorption, aggregation, and rapid RES clearance.

#### 6.1.1 Ligand removal LnNPs

Although quantitative *in vitro* IC_50_ or CC_50_ values for ligand-removed LnNPs are scarce in the existing literature, a substantial body of reports collectively indicates that these particles can induce aggregation-dependent cytotoxicity and significant ion leaching, thereby triggering oxidative stress and inflammatory responses [43,49,51,206]. For example, E. Wysokińska et al. reported that ligand-free NaGdF_4_:Yb/Er exhibited an IC_50_ of 26 μg/mL in NIH3T3 fibroblasts, but only 1.6 μg/mL in RAW264.7 macrophages [263]. Their data also showed that apoptosis was induced extensively at concentrations of 100 μg/mL and 10 μg/mL in the two cell types, respectively. Their results clearly demonstrate that bare NaGdF_4_:Yb/Er nanocrystals possess significant *in vitro* cytotoxicity. In recent years, studies using ligand-free LnNPs have also been reported with favorable toxicity data, but those were mostly obtained after further modification with liposomes or other coatings [49,51].

#### 6.1.2 Ligand oxidized LnNPs

To date, there have been no clear quantitative reports on the *in vitro* IC_50_ values of oxidized LnNPs, and their *in vivo* toxicity has not been systematically evaluated. However, the instability and potential toxicity of the decarboxylation products suggest a narrow safety window. Although Dai et al. reported that hydrazine-modified NaYF_4_:Yb/Tm LnNPs formed by further reaction of LnNPs with oxidized ligands did not exhibit obvious cytotoxicity against L929 fibroblasts at concentrations ranging from 3.125 μg/mL to 200 μg/mL, this finding does not represent a direct measurement of the ligand-oxidized LnNPs themselves and therefore offers limited reference value [17].

#### 6.1.3 Small molecule surface LnNPs

Small-molecule coatings such as tripolyphosphate (TPP) or citrate confer initial hydrophilicity but are prone to poor colloidal stability and rapid displacement by endogenous phosphates. For TPP-coated LaF_3_:Ce LnNPs, *in vitro* cytotoxicity assays in 3D pancreatic tumor spheroids yielded IC_50_ values of 2.7 mg/mL for PANC-1 cells and 3.0 mg/mL for MIA PaCa-2 cells [228]. Although TPP-coated NPs exhibited substantially higher cellular uptake (11.5-fold greater in spheroids), their *in vivo* safety profile was markedly inferior: intravenous administration at 200 mg/kg in mice led to rapid and irreversible accumulation in the liver and spleen (>90% ID/g at 1 h) with negligible clearance, ultimately causing significant organ toxicity. Consequently, no maximum tolerated dose (MTD) could be established for TPP-coated NPs due to persistent tissue retention.

In addition, Citrate is another important small-molecule ligand commonly used for hydrophilization modification. Liu et al. systematically evaluated the *in vitro* and *in vivo* toxicity of citrate-coated ultra-small UCNP (Cit-UCNPs, NaYF_4_:Yb/Gd, ∼5 nm) [264]. They found that cell viability exceeded 92% after 2 h exposure to 10–500 μg/mL Cit-UCNPs, and remained above 86% even after prolonged continuous exposure. Most other studies also report that citrate-coated LnNPs generally exhibit low to moderate cytotoxicity [[265], [266], [267]]. However, quantitative IC_50_/CC_50_ values for citrate-modified LnNPs are absent in the literature, most studies only qualitatively note good biocompatibility or no significant toxicity. *In vivo* MTD data are also scarce. This lack of data also precludes a reliable calculation of the safety margin for citrate-modified LnNPs.

### 6.2 PEGylated surfaces LnNPs

PEGylation, whether achieved via ligand exchange with PEG-phosphonate/PEG-carboxylate or through polymer encapsulation (e.g., DSPE-PEG), represents the most widely adopted strategy to improve the biocompatibility of LnNPs. The available toxicity data, though derived from different core compositions and cell models, consistently demonstrate that PEG coatings confer favorable safety profiles, with the magnitude of tolerance depending on particle size, core material, and administration regimen.

The most systematically characterized PEGylated system is LaF_3_:Ce (∼45 nm) coated with PEG. *In vitro*, these LnNPs exhibited an IC_50_ of 6.4 mg/mL against PANC-1 pancreatic spheroids and 5.4 mg/mL against MIA PaCa-2 spheroids, with >95% viability maintained at concentrations ≤2.5 mg/mL [228]. *In vivo*, intravenous administration of 200 mg/kg was well tolerated, with no body weight loss, behavioral changes, or mortality. Plasma biochemistry revealed only mild hepatic toxicity (elevated ALT/AST) after 14 days, while renal and muscular functions remained unaffected. The MTD was thus established at ≥200 mg/kg (i.v.), and progressive clearance (lanthanum levels decreased in all organs from 24 h to 14 days) contributed to a favorable safety margin of approximately 10- to 20-fold when compared to estimated therapeutic effective doses (10–20 mg/kg). Similar biocompatibility trends have been observed for PEG-coated NaYF_4_ UCNPs, although with different quantitative metrics. Cheng et al. reported that PEG-NaYF_4_ NPs (various sizes) at intravenous doses up to 50 mg/kg showed no acute toxicity in mice over 2 weeks, with histopathology revealing no major organ damage [249]. However, the exact MTD was not determined. In cell viability assays across multiple cell lines (HeLa, HEK293), IC_50_ values were >200 μg/mL (i.e., >0.2 mg/mL) for most PEGylated formulations, though the values varied with PEG chain length and terminal functionality [60,61]. Notably, these *in vitro* IC_50_ values are substantially lower than those for PEG-LaF_3_:Ce in 3D spheroids, likely reflecting differences in cell type (2D monolayer vs. 3D architecture), LnNPs core composition, and assay duration. In addition, in mouse models, PEG-modified BaGdF_5_:Yb/Er nanoprobes [38] and PEG-modified NaLuF_4_:Yb/Er [36] at doses of 10–20 mg/kg did not exhibit significant toxicity within 30 days. Although quantitative MTD data were not explicitly available, histological analysis provided some validation of safety at this dose.

Collectively, these data demonstrate that PEGylation consistently improves the safety profile of LnNPs, with MTD values ranging from ≥50 mg/kg (for NaYF_4_) to ≥200 mg/kg (for LaF_3_:Ce) depending on core composition and particle size. The *in vitro* IC_50_ values also span a wide range (from >0.2 mg/mL in 2D cultures to 5–6 mg/mL in 3D spheroids), underscoring the critical influence of the biological model and LnNPs physicochemical properties. Despite these variations, the overall evidence supports PEGylation as a robust strategy to achieve favorable safety margins for *in vivo* theranostic applications.

### 6.3 Inorganic surfaces LnNPs

Silicon coatings, due to their inertness, chemical stability, and effective isolation from lanthanide cores, provide quite robust safety characteristics.

Abdul Jalil and Zhang were the first to systematically evaluate the *in vivo* biocompatibility of silica-coated UCNPs [105]. Their results showed that the viabilities of BMSCs and myoblasts decreased after exposure to nanocrystals at concentrations of 1, 5, 10, 15, 25, 50, and 100 μg/mL for 12, 24, 36, and 48 h. By 48 h, BMSC viability dropped to 99.6%, 93.4%, 85.6%, 79%, 74.8%, 70.3%, and 66.8%, while skeletal myoblast viability decreased to 99.8%, 93.2%, 87.8%, 85.2%, 79.1%, 77.9%, and 68.2%, indicating considerable toxicity. This may be related to the thickness of the silica shell used in their material, since a thin silica coating cannot completely inhibit the dissolution of UCNPs [268], the silica shell thickness of the nanocrystals they synthesized was only 8 ± 1.5 nm [105]. In a recent study, Cynthia Kembuan et al. systematically compared the relationship between silica shell thickness and the toxicity of LnNPs [269]. Inductively coupled plasma optical emission spectrometry (ICP-OES) measurements showed that after 24 h of dispersion in water, the thin-shell (7 nm) sample released 0.97% of Y^3+^ ions, whereas the thick-shell (21 nm) sample released only 0.33%. For Yb^3+^, the thin shell released 0.41% and the thick shell released 0.15%. The 21 nm shell significantly extended the diffusion path of water molecules to the UCNP core compared to the 7 nm shell, and also hindered the outward leakage of Ln^3+^ and F^−^ ions from the core. The reduced ion release directly lowered oxidative stress and biochemical interference in cells. This was directly reflected in cytotoxicity tests: at the highest concentration (200 μg/mL), the cell viability of thin-shell aminated particles (UC@thin-NH_2_) was only about 51%, whereas that of thick-shell aminated particles (UC@thick-NH_2_) reached about 75%. Interestingly, an inverse phenomenon was observed at low concentration (12.5 μg/mL), where the viabilities of thin- and thick-shell particles were 110% and 95%, respectively—the thin shell even stimulated cell proliferation at low concentration, but toxicity became prominent at high concentration. Meanwhile, the cellular uptake of UC@thick-NH_2_ was actually higher than that of UC@thin-NH_2_, confirming that the main source of toxicity is the released ions. Even though more thick-shell particles were internalized, their thicker shell led to less ion release, thus causing less damage to cells.

Extending the silica coating concept to mesoporous architectures, formulations such as NaYF_4_:Yb/Er@mSiO_2_ [94,97] and LiYF_4_@mSiO_2_ [92] have been developed for drug delivery and PDT. While systematic *in vivo* toxicity studies remain limited, these particles are generally regarded as biocompatible due to the protective silica shell. *In vitro* cell viability assays in various cancer cell lines consistently demonstrate >80% viability at concentrations up to 200 μg/mL [92,94,270]. However, no MTD data are currently available for mSiO_2_-coated LnNPs, representing a knowledge gap that warrants future investigation, particularly given the potential for enhanced drug loading and sustained release from the mesoporous structure. In addition, other shelless enclosures also show good security, although they may have been further modified [122,126,160].

### 6.4 Comparative summary of toxicity profiles

A comprehensive analysis of various coating materials for LnNPs showed significant differences in reduced toxicity and biocompatibility. We summarized quantitative data from related studies and compared the effects of different coatings on cytotoxicity and overall biocompatibility (Table 13).

**Table 13:** Comparative summary of toxicity profiles of surface-engineered LnNPs.

Surface Strategy	Formulation	*In Vitro* Cytotoxicity	*In Vivo* MTD	Biocompatibility	Reference
**Ligand Removal (HCl)**	NaYF_4_:Yb/Er	IC_50_ (24 h) = 98.5 μg/mL	Not reported	Low	[267]
**Ligand Removal (BF^−^)**	NaYF_4_:Yb/Er	IC_50_ (24 h) = 395.6 - 423.9 μg/mL	Not reported	Low	[267]
**Small ligand**	TPP-LaF_3_:Ce	IC_50_ = 2.7–3.0 mg/mL (3D spheroids)	Not reported	Low	[228]
**Small ligand**	citrate- NaYF_4_:Yb/Er	IC_50_ = 563.4 μg/ml	Not reported	Moderate	[267]
**Alendronate-coated LnNPs**	Alendronate- NaYF_4_:Yb/Er	Not specified	Not specified	Low	[267]
**PEG**	PEG-LaF_3_:Ce	IC_50_ = 5.4–6.4 mg/mL	≥200 mg/kg (i.v.)	High	[228]
**PEG**	PEG-NaYF_4_	IC_50_ > 200 μg/mL	>50 mg/kg (i.v., no MTD reached)	High	[60,61,249]
**Silica-coated LnNPs (thick)**	NaYF_4_:Yb/Er@SiO_2_ (21 ± 3 nm)	IC_50_ > 200 μg/mL	Not specified	High	[269]
**Silica-coated LnNPs (thin)**	NaYF_4_:Yb/Er@SiO_2_ (7 ± 2 nm)	IC_50_ = 200 μg/mL	Not specified	Moderate	[269]

## 7 Conclusions and perspectives

The past decade has witnessed substantial progress in research on rare-earth-based inorganic nanomaterials. This review comprehensively covers the hydrophilic modification, surface functionalization, and applications in optical imaging and biotherapy of rare-earth-based inorganic luminescent nanomaterials. Future advances in this field will depend on achieving precise control over the composition and morphology of nanostructures, as well as advancing surface engineering strategies through interdisciplinary collaboration to address key challenges related to luminescence efficiency, biosafety, and clinical translation.

As an emerging class of luminescent biological probes that serve as alternatives to conventional molecular probes, Ln^3+^-doped inorganic luminescent nanomaterials have undergone substantial development in recent years, particularly in the context of biomedical applications [271]. Surface bioengineering has transformed LnNPs from a hydrophobic and bioinert state into a biomedical platform with favorable performance and diverse functionalities, serving as a bridge that links their unique optical properties to complex biological applications. However, factors such as composition, size, surface charge, shape, and surface modification play a role in the synthesis process and can influence cytotoxicity. In recent years, surface-modified LnNPs have shown potential in applications with high biosafety requirements, such as theranostics. These include the visualization guidance of phototherapy and radiotherapy, as well as the construction of personalized treatment strategies. Through precise surface engineering design, smart nanoplatforms based on LnNPs can achieve spatiotemporally controllable theranostics, which may reduce side effects while improving therapeutic outcomes. Although these nanobiological probes may not yet be fully ready for bioimaging or clinical bioanalysis, progress has been made across their fundamental chemistry and physics to biological applications, including controlled synthesis methodologies, surface modification chemistry, and proof-of-concept biological studies. Addressing the remaining challenges will be important to facilitate their clinical translation.

Regarding luminescence efficiency and stability, the high-frequency vibrational groups introduced by hydrophilic modification, together with complex core–shell structures, can lead to non-radiative energy loss. This often results in reduced luminescence efficiency, posing challenges for high-sensitivity biological imaging and therapy. Such quenching effects are relevant to many FL-based assays, where interactions between luminescent nanomaterials and aromatic structures or terminal polar groups such as –COOH, –SH, –COOR, –NH_2_, and –OH can contribute to emission attenuation. Therefore, developing next-generation surface engineering and energy level regulation strategies remains a priority to enhance the luminescence brightness and photostability of LnNPs.Numerous published studies have highlighted advances in luminescent LnNPs, driven by collaborative efforts across research teams working to develop nanomaterials with diverse morphologies and functionalities. Nevertheless, efforts continue toward the synthesis of both existing and new LnNPs through innovative and improved methodologies, with the aim of achieving enhanced luminescence, improved performance, and greater suitability for complex biological environments. To date, Ln^3+^ ions such as Er^3+^, Ho^3+^, Yb^3+^, Tm^3+^, and Nd^3+^ have been explored as sensitizers, operating across a broad wavelength range. Such developments have enhanced UCL features and expanded the range of options for extended applications. In practice, however, further progress remains limited. Future efforts will require more systematic and reproducible studies, along with practical energy transfer pathways to access additional activators and new host materials featuring advanced composition and structural design, in order to advance the luminescence efficiency of LnNPs.

For biological applications, ensuring colloidal stability in polar solutions is essential, as biomacromolecules readily interact with functional groups on LnNPs surfaces. For instance, nonspecific leakage of Ln^3+^ ions or therapeutic payloads during *in vivo* delivery may reduce therapeutic efficacy and potentially induce toxicity due to unintended biodistribution. Developing surface modification layers that combine favorable biocompatibility, long-term colloidal stability, and efficient active targeting capabilities is therefore important for optimizing treatment outcomes and minimizing off-target effects.

For precision therapy, stimulus-responsive systems such as those triggered by pH, enzymes, or ROS must be well matched to the tumor microenvironment. However, limitations in the sensitivity and control precision of external stimulation systems, along with the limited specificity of endogenous responses, can constrain therapeutic efficacy. Additionally, the emergence of personalized nanomedicine introduces another layer of complexity. These therapies, often customized for individual patients using genetic, proteomic, or metabolic data, challenge conventional regulatory concepts of batch uniformity and reproducibility [272]. Personalized formulations may be manufactured at micro‐scale volumes, making traditional quality control testing and pharmacovigilance protocols difficult to implement. Future regulatory frameworks must be designed to evaluate such products using flexible, risk‐based approaches that ensure patient safety without imposing impractical constraints on innovation. Moreover, personalized nanomedicines frequently rely on sensitive personal health information, particularly genetic data. This necessitates strict regulatory oversight regarding data privacy, ownership, and ethical use. Regulatory strategies must include robust cybersecurity measures, consent procedures, and cross‐jurisdictional data governance models that safeguard patient rights while enabling scientific progress [273].

The complexity of surface modification processes, variability in batch-to-batch reproducibility, and the absence of standardized characterization and quality control systems represent significant barriers to the clinical translation of LnNPs [274,275]. However, rare-earth luminescent materials still hold promise for clinical applications across various biomedical fields, yet several challenges remain to be addressed. A systematic approach is needed to tackle issues related to scalability, targeting efficiency, clinical validation, biocompatibility, and ethical considerations. In parallel with these efforts, recent advances in green nanotechnology, including biosurfactant-based functional nanoemulsions [276], have provided environmentally sustainable alternatives for surface engineering. By incorporating green synthesis principles into the hydrophilic modification and bio-functionalization of LnNPs, it becomes possible to reduce both biological and environmental toxicity while still preserving the desired physicochemical properties [277]. Such efforts are essential for translating laboratory research on LnNPs into routine clinical practice.

Looking ahead, the integration of knowledge across disciplines will be important for revealing new functions of rare-earth-doped nanomaterials in emerging research fields. Controlled synthesis of LnNPs has long been a significant challenge in rare-earth-based inorganic luminescent nanomaterials, and future efforts in synthesis and surface engineering are expected to address these challenges through interdisciplinary collaboration and innovation. A new generation of surface modification strategies, informed by computational chemistry, machine learning [[278], [279], [280]], theoretical simulation, and high-throughput screening of materials, surface ligands, and biological interfaces, may guide the construction of surface coatings with high luminescence efficiency, long-term stability, precise biological functionality, and intelligent responsiveness. In this context, intelligent rare-earth-based luminescent materials that exhibit dynamic responses to external stimuli (e.g., light, temperature, pH, or redox changes) have recently been demonstrated at the molecular level. Translating such smart responsiveness to LnNP surface coatings through rational ligand design or stimuli-cleavable linkers could enable on-demand modulation of imaging and therapeutic functions, representing a key direction for next-generation theranostic nanoplatforms [281].

Efforts to develop reliable biological detection agents and fluorescent molecular probes for molecular biology, as well as LnNPs-based nanocontrast agents and nanomedicines, are essential for advancing these nanoprobes toward clinical use as standard imaging reagents and safe, effective therapeutics. A handful of Ln-based compounds have successfully advanced through clinical trials. Currently, Gd-based complexes, especially heptodotic acid (Artirem®), highvaleric acid (Magnevist®), and hepttodopicone (Elucirem**^TM^**), have been widely used as MRI contrast agents for cancer imaging. At the same time, many promising Ln compounds are still under clinical evaluation in Phase I, II, and III [282,283].

To date, safety evaluations of LnNPs have been conducted primarily in small animal models such as mice. Until recently, Gadoquatrane, a novel gadolinium-based contrast agent (GBCA) currently in Phase III clinical development, exhibited pharmacokinetic characteristics similar to macrocyclic GBCA in crab-eating monkeys: rapid renal excretion, low tissue retention, and high metabolic stability. Compared to existing GBCAs, it does not increase the risk of long-term gadolinium residue in tissues [284]. However, translating these findings into larger-scale animal models remains challenging. For example, Antonio Petrini and colleagues recently reported that LnNPs used for diagnosis can persist for a long time in animals [285]. For example, for Gd, 1.5–2.5 μg Gd/g tissue can still be detected in the cerebellum 35 months after injection in dogs, while for Swiss alpine sheep, Ten weeks after injection, the highest concentrations were found in the kidneys (502 ng/g) and liver (445 ng/g), while the deep cerebellar nuclei were lower (30 ng/g), with no histopathological changes observed. Although lanthanides are low in content and considered to be less toxic, they have a significant impact on biological systems. Although recent studies focusing mainly on mice and rats have revealed some pathways by which these elements influence, their complex interactions with mammalian biology remain largely underexplored. LnNPs have various effects on cell viability and function, ranging from cytotoxicity to protective effects, depending on concentration, cell type, and environmental conditions. This dual behavior suggests potential therapeutic applications, especially in cancer treatment [252,286].

Advancing basic biomedical research and its clinical applications will require collaborative efforts across disciplines. For future multimodal bioanalysis and cancer therapy, integrating emerging imaging modalities such as CT and PET with established luminescence or magnetic resonance imaging techniques based on rare-earth-doped inorganic nanobiomarkers represents a promising direction. The clinical trials of Ln-based compounds, particularly focusing on the application of terbium isotopes such as ^152^Tb and ^161^Tb, demonstrate groundbreaking advancements in nuclear medicine for cancer diagnosis and therapy. Promising compounds include ^152^Tb-DOTATOC, which was clinically tested as a diagnostic agent in PET/CT imaging in a groundbreaking first-in-human clinical study [287]. A notable first-in-humans study evaluated the feasibility and safety of ^161^Tb-DOTATOC in two patients with metastatic neuroendocrine neoplasms (NENs). The compound was administered to a 35-year-old male with a nonfunctional paraganglioma and a 70-year-old male with a functional NEN of the pancreatic tail. Imaging outcomes revealed high-quality SPECT/CT images, enabling precise visualization of metastases, including those in the liver and bones. The biodistribution data indicated uptake in key organs like the liver, kidneys, and spleen, and the procedure confirmed the utility of ^161^Tb for both imaging and potential therapeutic applications. Importantly, no adverse events were reported.

More comprehensive studies may provide richer, higher-quality preclinical data to support the development of LnNPs as next-generation diagnostic and therapeutic drug classes.

**Table:** Table of Abbreviations

Abbreviation	Full Form
LnNPs	Lanthanide Nanoparticles
NIR	near-infrared region
NIR-II	Near-infrared region II
FL	fluorescence
UCL	up-conversion luminescence
Ln^3+^	lanthanides
LBL	layer-by-layer
OA	Oleic acid
OM	Oleylamine
NPs	Nanoparticles
PAA	Poly(acrylic acid)
AEP	2-Aminoethyl dihydrogen phosphate
UCNPs	Up-conversion Nanoparticles
FRET	Förster resonance energy transfer
PVP	Polyvinylpyrrolidone
PEG	Polyethylene glycol
DOX	Doxorubicin
NOBF_4_	Nitrosonium tetrafluoroborate
DMF	N, N-dimethylformamide
PEI	Polyethylenimine
TEOS	Tetraethyl orthosilicate
APTES	(3-Aminopropyl)triethoxysilane
mSiO_2_	Mesoporous silica
PBS	Phosphate-buffered saline
EDC	1-Ethyl-3-(3-dimethylaminopropyl)carbodiimide
NHS	N-Hydroxysuccinimide
FA	Folic acid
CTPP	cell penetrating peptide
CuAAC	Copper(I)-catalyzed azide–alkyne cycloaddition
SPAAC	Strain-promoted azide–alkyne cycloaddition
DBCO	dibenzocyclooctene
ICG	Indocyanine green
MOF	metal–organic framework
MRI	Magnetic resonance imaging
SERS	surface-enhanced Raman scattering
TPP	tripolyphosphate
Cur	Curcumin
CAZ	Ceftazidime
PLL	poly-L-lysine
ROS	Reactive oxygen species
^1^O_2_	singlet oxygen
PDT	Photodynamic therapy
PTT	Photothermal therapy
AIE	aggregation-induced emission
TQ	photosensitizer
SPECT	Single photon emission computed tomography
CT	Computed tomography
PET	Positron emission tomography
ADME	Absorption, distribution, metabolism, and excretion
RES	Mononuclear phagocyte system
MTD	Maximum tolerated dose
IC_50_	Half maximal inhibitory concentration
CC_50_	Half maximal cytotoxic concentration
ALT	Alanine aminotransferase
AST	Aspartate aminotransferase

## CRediT authorship contribution statement

**Xuan Tan:** Conceptualization, Data curation, Formal analysis, Investigation, Visualization, Writing – original draft. **Yunqiu Zhang:** Investigation, Project administration, Writing – original draft. **Shuping Wu:** Investigation. **Dengyu Ma:** Investigation. **Jinyu Yan:** Investigation. **Jinwei Lu:** Investigation. **Shikai Lin:** Investigation. **Shiyan Li:** Investigation. **Zhexin Hong:** Investigation. **Shihui Jiang:** Resources, Supervision, Writing – review & editing. **Guowei Li:** Conceptualization, Funding acquisition, Resources, Supervision, Writing – review & editing.

## Declaration of competing interest

The authors declare that they have no known competing financial interests or personal relationships that could have appeared to influence the work reported in this paper.

## Data Availability

No data was used for the research described in the article.
